# Sleep Deprivation-Induced Oxidative Stress in Rat Models: A Scoping Systematic Review

**DOI:** 10.3390/antiox12081600

**Published:** 2023-08-11

**Authors:** Vlad Sever Neculicioiu, Ioana Alina Colosi, Carmen Costache, Dan Alexandru Toc, Alexandra Sevastre-Berghian, Horațiu Alexandru Colosi, Simona Clichici

**Affiliations:** 1 Department of Microbiology, “Iuliu Hatieganu” University of Medicine and Pharmacy, 400349 Cluj-Napoca, Romania; 2 Department of Physiology, “Iuliu Hatieganu” University of Medicine and Pharmacy, 400006 Cluj-Napoca, Romania; 3 Division of Medical Informatics and Biostatistics, Department of Medical Education, “Iuliu Hatieganu” University of Medicine and Pharmacy, 400349 Cluj-Napoca, Romania

**Keywords:** sleep deprivation, sleep, oxidative stress, stress, glutathione, GSH, GSSG, catalase, CAT, superoxide dismutase, SOD, nitric oxide, NOx, lipid peroxidation, MDA, rat

## Abstract

Sleep deprivation is highly prevalent in the modern world, possibly reaching epidemic proportions. While multiple theories regarding the roles of sleep exist (inactivity, energy conservation, restoration, brain plasticity and antioxidant), multiple unknowns still remain regarding the proposed antioxidant roles of sleep. The existing experimental evidence is often contradicting, with studies pointing both toward and against the presence of oxidative stress after sleep deprivation. The main goals of this review were to analyze the existing experimental data regarding the relationship between sleep deprivation and oxidative stress, to attempt to further clarify multiple aspects surrounding this relationship and to identify current knowledge gaps. Systematic searches were conducted in three major online databases for experimental studies performed on rat models with oxidative stress measurements, published between 2015 and 2022. A total of 54 studies were included in the review. Most results seem to point to changes in oxidative stress parameters after sleep deprivation, further suggesting an antioxidant role of sleep. Alterations in these parameters were observed in both paradoxical and total sleep deprivation protocols and in multiple rat strains. Furthermore, the effects of sleep deprivation seem to extend beyond the central nervous system, affecting multiple other body sites in the periphery. Sleep recovery seems to be characterized by an increased variability, with the presence of both normalizations in some parameters and long-lasting changes after sleep deprivation. Surprisingly, most studies revealed the presence of a stress response following sleep deprivation. However, the origin and the impact of the stress response during sleep deprivation remain somewhat unclear. While a definitive exclusion of the influence of the sleep deprivation protocol on the stress response is not possible, the available data seem to suggest that the observed stress response may be determined by sleep deprivation itself as opposed to the experimental conditions. Due to this fact, the observed oxidative changes could be attributed directly to sleep deprivation.

## 1. Introduction

Sleep is a highly conserved fundamental aspect observed across the animal kingdom, playing a critical role in maintaining homeostasis. Sleep deprivation (SD) refers mainly to quantitative or qualitative alterations of normal sleep. SD seems to be intrinsically linked to the modern technological world and lifestyle. Decreases in sleep duration or quality can be seen in all age groups, with significant variations determined by a multitude of factors [1]. While SD is certainly common, there is no current agreement on the extent of this phenomenon to epidemic proportions [2]. In humans, SD determines a multitude of negative health effects ranging from daytime sleepiness to an association with multiple chronic conditions such as obesity, diabetes, mental health disorders, cardiovascular disease and neurodegenerative disorders [2,3,4]. 

Sleep is a universal phenomenon in most animals. Even though the first experimental evidence regarding the importance of sleep dates back to the 19th century [5], multiple unknowns still persist regarding the nature of sleep, particularly regarding its underlying functions. While several theories attempt to explain the roles of sleep (such as the inactivity theory, energy conservation theory, restoration theory and brain plasticity theory, among others), it is commonly agreed that the roles of sleep are best explained through an integration of these theories [6]. Furthermore, an emerging perspective describes sleep as a consequence of metaregulation. Through this lens, the beneficial role of sleep can be explained by considering sleep as a state of adaptive inactivity or as a default state of cerebral networks [7].

A further theory regarding the role of sleep was first proposed by Reimund in 1994, hypothesizing that sleep may play an antioxidant role in the brain and peripheric organs [8]. Initially, this theory was disregarded due to the frequently contradictory evidence in the literature regarding animal models of SD and oxidative stress. Nevertheless, recent findings appear to validate the antioxidant functions of sleep and the occurrence of oxidative stress after SD in both paradoxical sleep deprivation (PSD/REM sleep) and total sleep deprivation (TSD) protocols in rodent models [9]. Additionally, a multifaceted reciprocal association between sleep and oxidative stress has been proposed and seems to be supported by recent results: sleep may act as a protective mechanism against oxidative stress, while at the same time, a certain level of oxidative stress is required to initiate sleep [9,10]. While the behavioral effects of sleep deprivation (such as low mood, anxiety, memory alterations etc.) have been largely characterized before, numerous unknowns remain regarding the molecular changes underlying these effects. Furthermore, it is believed that the molecular and immune alterations resulting from SD, such as oxidative stress and inflammation, may serve as driving factors for the development of chronic pathologies linked to insufficient sleep [4,11]. 

Oxidative stress can be defined as “an imbalance between oxidants and antioxidants in favor of the oxidants, leading to a disruption of redox signaling and control and/or molecular damage” [12]. Reactive oxygen species (ROS) span a spectrum of roles depending on their concentration. At low levels (oxidative eustress), ROS encompass crucial functions in multiple physiological processes such as redox signaling, cell death, and immune function. However, at higher concentrations, ROS can be damage-inducing (oxidative distress) [13,14]. Overall, redox homeostasis is maintained through multiple oxidative stress responses following activation by multiple molecular redox switches [13]. The imbalance between oxidants and antioxidants observed in oxidative stress can occur due to various factors, including increased reactive oxygen species (ROS) production, antioxidant enzyme inactivation or increased antioxidant consumption [14]. The excess ROS formed during oxidative stress (superoxide anion, hydrogen peroxide, hydroxyl radical, peroxyl radicals etc.) is counterbalanced by both enzymatic (Superoxide dismutase, Catalase, Glutathione peroxidase and transferase) and nonenzymatic antioxidants (Glutathione, vitamins, etc.). The roles and determination methods of the major antioxidants are relatively well-known and have been previously reviewed [12,14,15]. The unbalanced increased oxidant load can lead to an accumulation of damage, activating multiple programs such as autophagy, mitophagy, apoptosis, necroptosis, and ferroptosis [13]. Furthermore, increased ROS production may determine changes in DNA structure, induce alterations in lipids and proteins, trigger the activation of transcription factors, impact the synthesis of inflammatory cytokines and influence signal transduction pathways [15]. Oxidative damage to lipids generates a wide range of toxic compounds, ranging from lipid hydroperoxides to Malonaldehyde (MDA) and 4-Hydroxynonenal (4-HNE) [16]. These products can further induce protein and DNA damage by cross-linking or denaturation [15,17]. Nitric oxide (NO) exhibits intricate physiological and pathological functions ranging from signaling, inflammation, vascular regulation, regulation of sleep–wake cycles and oxidative damage. NO synthesis is mediated by three enzyme isoforms, each possessing specific localizations and distinct roles: neuronal nitric oxide synthase (nNOS), inducible nitric oxide synthase (iNOS) and endothelial nitric oxide synthase (eNOS) [18]. NO plays an intricate role in relation to oxidative stress. While increased ROS can decrease NO availability through NO scavenging, especially in the cardiovascular system [19], dysregulated nitric oxide can be implicated in oxidative/nitrosative stress, which could further induce oxidative damage in proteins, lipids and DNA [20,21]. 

A previous systematic review on the relationship between sleep deprivation and oxidative stress has been published by Villafuerte et al. [9], presenting evidence for the antioxidant role of sleep in both brain and non-brain areas. However, multiple unknowns remain regarding this relationship. We performed a scoping review in order to systematically identify and evaluate the newly available research conducted on this topic. The main goals of this review were to evaluate and attempt to clarify the relationship between sleep deprivation and oxidative stress in experimental studies performed on rat models, to provide a timeline of oxidative changes determined by sleep deprivation and to identify and address any remaining knowledge gaps regarding this relationship.

## 2. Materials and Methods

The methods used in this review were designed and based on the 22-item Preferred Reporting Items for Systematic Reviews and Meta-Analyses extension for Scoping Reviews (PRISMA-ScR) [22].

A scoping systematic review of the literature was performed regarding oxidative changes in experimental sleep-deprived rat models. The searches were performed between 21 and 23 February 2023 in three databases PubMed, Web of Science and Cochrane Library, for articles published between 1 January 2015 and 1 October 2022. The following simplified search algorithm was adapted for each database: (sleep OR sleep deprivation) AND (oxidative stress) AND (rat) AND (publication date 1 January 2015–1 October 2022) NOT (sleep apnea OR neurodegenerative disease). Search results were imported into Endnote 20 (IBM–Clarivate) for management. Title and Abstract screening and full-text evaluation were performed by two researchers (V.S.N. and I.A.C.), and discrepancies were resolved by consensus and oversight from C.C., H.A.C. and S.C. A data charting form was initially piloted and developed by two authors (V.S.N. and I.A.C.) and updated as needed. The following data were extracted from each included study: sleep deprivation protocol and duration, type of control group, subject characteristics (rat breed, age and sex), serum cortisol/corticosterone, oxidative stress parameters (Reduced Glutathione–GSH, Oxidized Glutathione–GSSG, GSH/GSSG ratio, Catalase–CAT, Superoxide dismutase–SOD, Nitric Oxide and Nitric Oxide enzymes), Lipid peroxidation. Data extraction was performed independently by two authors (V.S.N. and I.A.C.). 

Comparisons were sought between a control group not exposed to sleep deprivation and a group that was sleep deprived without any other intervention or drug administration. We included experimental sleep deprivation studies performed on rats, using any type of sleep deprivation protocol that included at least one of the previously mentioned oxidative stress parameters. All measurement types were included (enzyme activity, ELISA, Western Blot, Immunohistochemistry, Immunofluorescence, gene expression, etc.).

No automation tools were employed during the search, screening, full-text evaluation or data extraction steps of the review. A limited methodological and bias evaluation was performed.

The included studies were grouped by type of SD protocol (PSD or TSD), by anatomical region in which the oxidative stress measurements were performed and by the length of the SD duration. In some cases, studies that performed oxidative stress measurements in multiple body sites were presented in multiple tables according to the previously detailed grouping.

**Figure 1:**
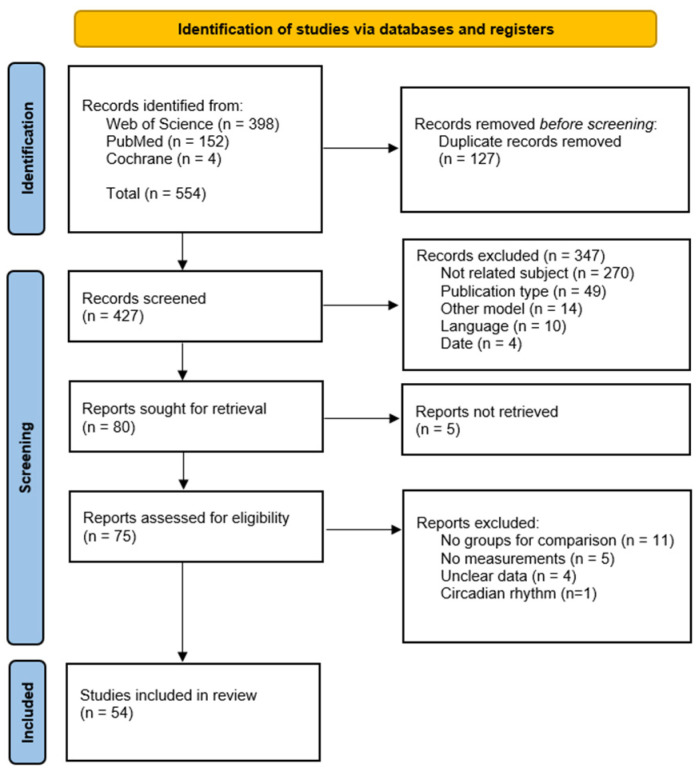
Study inclusion flowchart [23].

A comprehensive overview of the methods can be found in Supplementary File S1. A flowchart presenting the study inclusion process is presented in Figure 1. 

## 3. Results

A total of 54 studies were included in the qualitative analysis. Multiple sleep deprivation protocols were used, such as MSP (multiple small platforms), CP (classical platform/inverted flowerpot), DOW (Disk over water), GH (Gentle handling) and ASD (automated sleep deprivation). Comprehensive descriptions of the mentioned sleep deprivation protocols and respective control groups have been previously reviewed [9,24,25].

Most of the included studies employed paradoxical sleep deprivation (PSD) protocols (87%, *n* = 47), while the others used total sleep deprivation (TSD) protocols (13%, *n* = 7). 

The studies that evaluated PSD employed the following SD protocols: Multiple small platforms (68.5%, *n* = 37/54) and Classical Platform/Inverted flowerpot (18.5%, *n* = 10/54).

The studies that evaluated TSD employed the following SD protocols: Disk over water (5.5%, *n* = 3), Gentle handling (3.7%, *n* = 2) and Automated sleep deprivation (3.7%, *n* = 2). 

The main results of the review are presented in Table 1, Table 2, Table 3 and Table 4 for paradoxical sleep deprivation in the hippocampus, cortex and other brain areas, serum, other non-brain areas, respectively; Table 5 and Table 6 for total sleep deprivation in brain and non-brain areas and Table 7 for gene expression in both PSD and TSD protocols. The full-length tables are available in Supplementary File S1.

Overall, only a limited number of studies (11.1%, *n* = 6/54) reported a lack of significant changes in any of the evaluated parameters compared to their respective control group [26,27,28,29,30,31]. Conversely, the overwhelming majority of results point to either increases or decreases in at least one parameter associated with oxidative stress. Remarkably, a notable pattern seems to emerge across a majority of studies whereby the examined parameters exhibit relatively consistent changes, with either a lack of changes, reductions in some parameters such as antioxidants/antioxidant enzymes (GSH, GSH/GSSG ratio, GPx, SOD, CAT) or increases in others (GSSG and lipid peroxidation). Only three of the examined studies seem to present contradicting results, with increases in the antioxidant GSH [32] or in the antioxidant enzyme SOD [33,34]. 

The stress response was evaluated in a limited number of studies through the measurement of serum Cortisol/Corticosterone (18.5%, *n* = 10/54), almost exclusively in PSD protocols employing the MSP paradigm. In most of these studies, serum Cortisol/Corticosterone was significantly increased in the sleep deprivation group compared to the control group in both continuous and sleep restriction protocols [34,35,36,37,38,39,40,41]. Only two studies reported no significant changes in this parameter [42,43]. A comprehensive comparison between studies that determined serum stress hormones is available in Table 8.

**Table 1:** Paradoxical sleep deprivation in the hippocampus.

Reference	SD Protocol	SD Duration	Rat Breed, Sex, Age	Oxidative Stress Marker	Results
[27]	MSP	48 h	Wistar, Male, PND 28	LP	LP (-)
[44]	MSP	48 h	Wistar, Male, Adult	GSH, NOx, LP	GSH (↓), NOx (↑), LP (-)
[26]	MSP	72 h	Wistar, Male, Adult	GSH, LP	GSH (-), LP (-)
[45]	MSP	72 h	Wistar, Male	LP	LP (↑)
[46]	MSP	72 h	Wistar, Male, Adult	GSH, SOD, LP	GSH (↓), SOD (↓), LP (↑)
[47]	MSP	72 h	Wistar, Male, Adult	LP	LP (↑)
[31]	MSP	24, 48, 72 h	Wistar, Male, Adult	GSH, GPx, CAT, SOD, LP	GSH (-), GPx (-), CAT (-), SOD (-), LP (-)
[48]	CP	4 days	Wistar, Male, 5 weeks	LP	LP (↑) *
[49]	MSP	5 days	Sprague Dawley, Male and Female, 6 weeks	GSH/GSSG ratio, GPX, CAT, SOD, LP	GSH/GSSG ratio (↓), GPX (-), CAT (↓), SOD (↓), LP (↑)
[38]	MSP	5 days	Sprague Dawley, Male, 48 weeks	NOx	NOx (↑)
[50]	CP	6 days (48 h SD, 48 h Srec, 48 h SD, 48 h Srec, 48 h SD)	Wistar, Male, Adult	CAT, LP	CAT (-), LP (↑)
[43]	MSP	21 days (18 h/day) 21 days (18 h/day) + 5 days Srec 21 days (18 h/day) + 21 days Srec	Sprague Dawley, Male, 12–16 weeks	GSH/GSSG ratio, LP	GSH/GSSG ratio (↓), LP (↑) GSH/GSSG ratio (↓), LP (↑) GSH/GSSG ratio (↓), LP (-)
[51]	MSP	4 weeks (8 h/day)	Wistar, Male, Adult	GSH, GSSG, GSH/GSSG ratio, GPx, CAT	GSH (-), GSSG (↑), GSH/GSSG ratio (↓), GPx (↓), CAT (↓)
[52]	MSP	4 weeks (8 h/day)	Wistar, Male, Adult	GSH, GSSG, GSH/GSSG ratio, GPx, CAT	GSH (-), GSSG (↑), GSH/GSSG ratio (↓), GPx (↓), CAT (↓)
[53]	MSP	4 weeks (8 h/day)	Wistar, Male, Adult	GSH, GSSG, GSH/GSSG ratio, GPx, CAT, SOD, LP	GSH (-), GSSG (↑), GSH/GSSG ratio (↓), GPx (↓), CAT (↓), SOD (↓), LP (-)
[54]	MSP	6 weeks (8 h/day)	Wistar, Male, 8–10 weeks	GSH, GSSG, GSH/GSSG ratio, GPx, CAT, SOD, LP	GSH (-), GSSG (↑), GSH/GSSG ratio (↓), GPx (↓), CAT (↓), SOD (↓), LP (-)
[55]	MSP	6 weeks (8 h/day)	Wistar, Male, Adult	GSH, GSSG, GSH/GSSG ratio, GPx, CAT, SOD, LP	GSH (-), GSSG (↑), GSH/GSSG ratio (↓), GPx (↓), CAT (↓), SOD (↓), LP (-)
[56]	MSP	6 weeks (8 h/day)	Wistar, Male, Adult	GSH, GSSG, GSH/GSSG ratio, GPx, CAT, SOD	GSH (-), GSSG (↑), GSH/GSSG ratio (↓), GPx (↓), CAT (↓), SOD (↓)
[57]	MSP	6 weeks (8 h/day)	Wistar, Male, Adult	GSH, GSSG, GSH/GSSG ratio, CAT, SOD, LP	GSH (-), GSSG (↑), GSH/GSSG ratio (↓), CAT (↓), SOD (↓), LP (-)
[58]	MSP	8 weeks (8 h/day)	Wistar, Male, Adult	GSH, GSSG, GSH/GSSG ratio, GPx, CAT, SOD, LP	GSH (-), GSSG (↑), GSH/GSSG ratio (↓), GPx (↓), CAT (↓), SOD (-), LP (-)
[59]	MSP	8 weeks (8 h/day)	Wistar, Male, Adult	GSH, GSSG, GSH/GSSG ratio, GPx, CAT	GSH (-), GSSG (↑), GSH/GSSG ratio (↓), GPx (↓), CAT (↓)

SD: sleep deprivation; MSP: multiple small platforms; CP: classical platform/inverted flowerpot; Srec: sleep recovery; PND: postnatal day; GSH: Reduced Glutathione; GSSG: Oxidized Glutathione; GSH/GSSG ratio: Reduced Glutathione/Oxidized Glutathione ratio; GPx: Glutathione peroxidase; CAT: Catalase; SOD: Superoxide dismutase; NOx: Nitric oxide; LP: Lipid peroxidation; *: dentate gyrus, hippocampus; “↑/↓”: significantly increased/decreased; “-”: not significantly increased or decreased.

**Table 2:** Paradoxical sleep deprivation in cortex and other brain areas.

Reference	SD Protocol	SD Duration	Rat Breed, Sex, Age	Anatomical Site	Oxidative Stress Marker	Results
[28]	MSP	48 h	Wistar, Male, Adult	Cortex	GSH, NOx, LP	GSH (-), NOx (-), LP (-)
[26]	MSP	72 h	Wistar, Male, Adult	Prefrontal cortex	GSH, LP	GSH (-), LP (-)
[45]	MSP	72 h	Wistar, Male	Forebrain cortex	LP	LP (↑)
[47]	MSP	72 h	Wistar, Male	Forebrain cortex	LP	LP (↑)
[48]	CP	4 days	Wistar, Male, 5 weeks	Cortex	LP	LP (↑)
[60]	MSP	7 days	Wistar, Male, 8 weeks	Cortex	GSH, GPx, CAT, SOD, LP	GSH (↓), GPx (↓), CAT (↓), SOD (↓), LP (↑)
[26]	MSP	72 h	Wistar, Male, Adult	Cerebellum, Brainstem	GSH, LP	GSH (-), LP (-)
[36]	MSP	72 h	Sprague Dawley, Adult	Amygdala	SOD, LP	SOD (↓), LP (↑)
[61]	CP	72 h	Sprague Dawley, Male, 8–10 weeks	Thalamus	GSH, CAT, SOD, LP	GSH (↓), CAT (↓), SOD (↓), LP (↑)
[62]	MSP	5 days	Sprague Dawley, Male and Female, Adult	Whole brain	CAT, SOD, LP	CAT (↓), SOD (↓), LP (↑)
[63]	CP	6 days	Wistar, Male, Adult	Locus coeruleus	GSH	GSH (↓)

SD: sleep deprivation; MSP: multiple small platforms; CP: classical platform/inverted flowerpot; GSH: Reduced Glutathione; GPx: Glutathione peroxidase; CAT: Catalase; SOD: Superoxide dismutase; NOx: Nitric oxide; LP: Lipid peroxidation; “↑/↓”: significantly increased/decreased; “-”: not significantly increased or decreased.

**Table 3:** Paradoxical sleep deprivation in serum/plasma.

Reference	SD Protocol	SD Duration	Rat Breed, Sex, Age	Oxidative Stress Markers	Results
[39]	MSP	20 h	ns	LP	LP (↑)
[35]	MSP	24 h	Long-Evans, Male, Old	LP	LP (↑)
[64]	CP	24, 36, 48 h	Sprague Dawley, Male, 6 months	SOD, LP	SOD (↓), LP (↑)
[65]	CP	48 h	Sprague Dawley, Male, 6 weeks	SOD, LP	SOD (↓), LP (↑)
[17]	MSP	72 h	Wistar, Male, 10 weeks	NOx	NOx (↓)
[36]	MSP	72 h	Sprague Dawley, Adult	SOD, LP	SOD (↓), LP (↑)
[40]	MSP	5 days	Wistar, Male	LP	LP (↑)
[39]	MSP	5 days (20 h/day) 5 days (20 h/day) + 5 days Srec	ns	LP	LP (↑) LP (-)
[50]	CP	6 days (48 h SD, 48 h Srec, 48 h SD, 48 h Srec, 48 h SD)	Wistar, Male, Adult	CAT, LP	CAT (-), LP (-)
[66]	MSP	7 days (20 h/day) and 7 days continuous	Wistar, Male	CAT, SOD, LP	CAT (-/↓), SOD (-), LP (↑)
[67]	MSP	7 days	Wistar, Male	GPx, SOD	GPx (↓), SOD (↓)
[30]	MSP	21 days (18 h/day)	Wistar, Male, Adult	eNOS	eNOS (-)

SD: sleep deprivation; MSP: multiple small platforms; CP: classical platform/inverted flowerpot; Srec: sleep recovery; GPx: Glutathione peroxidase; CAT: Catalase; SOD: Superoxide dismutase; NOx: Nitric oxide; eNOS: Endothelial Nitric Oxide Synthase; LP: Lipid peroxidation; ns: not specified; “↑/↓”: significantly increased/decreased; “-”: not significantly increased or decreased.

**Table 4:** Paradoxical sleep deprivation in other non-brain sites.

Reference	SD Protocol	SD Duration	Rat Breed, Sex, Age	Anatomical Site	Oxidative Stress Markers	Results
[17]	MSP	72 h	Wistar, Male, 10 weeks	Testes, Epididymis	GSH, GPx, CAT, SOD, LP	GSH (↓), GPx (↓), CAT (↓), SOD (↓), LP (↑)
[40]	MSP	5 days	Wistar, Male	Testes	GSH, GPx, LP	GSH (↓), GPx (↓), LP (↑)
[34]	MSP	14 days (20 h/day)	Sprague Dawley, Male, 12 weeks	Testes	GSH, CAT, SOD, LP	GSH (↓), CAT (↓), SOD (↑), LP (↑)
[41]	MSP	21 days (18 h/day)	Wistar, Male, Peripubertal	Testes	GSH, GSSG, GSH/GSSG ratio, LP	GSH (-), GSSG (-), GSH/GSSG ratio (-), LP (↑)
[32]	MSP	21 days (18 h/day)	Wistar, Male, Peripubertal	Epididymis caput, cauda	GSH, LP	GSH (↑), LP (↑)
[33]	MSP	4 days 8 days 8 days + 20 days Srec	Wistar, Male, 3 months	Liver, Pancreas	CAT, SOD, LP	CAT (-), SOD (-/↑), LP (-/↑) CAT (↓/-), SOD (↓/-), LP (↑/-) CAT (-), SOD (↓/-), LP (↑)
			Wistar, Male, 14 months	Liver, Pancreas	CAT, SOD, LP	CAT (↓/-), SOD (-), LP (-) CAT (↓/-), SOD (-/↑), LP (-/↑) CAT (-), SOD (-/↑), LP (-)
[68]	MSP	21 days (14 h/day)	Wistar, Male, Adult	Liver	SOD, LP	SOD (↓), LP (↑)
[69]	CP	21 days (18 h/day) 21 days (22 h/day)	Wistar, Male, Adult	Liver	GPx, SOD, LP	GPx (-), SOD (-), LP (-) GPx (-), SOD (↓), LP (↑)
[70]	CP	72 h 72 h + 72 h Srec	Sprague Dawley, Male, 8–10 weeks	Aorta	SOD, LP	SOD (↓), LP (↑) SOD (-), LP (-)
[71]	CP	5 days	Sprague Dawley, Male, 24 weeks	Aorta	NOx, eNOS, p-eNOS	NOx (↓), eNOS (-), p-eNOS (↓)
[66]	MSP	7 days (20 h/day) and 7 days continuous	Wistar, Male	Saliva Submandibular	CAT, SOD, LP	CAT (-), SOD (-), LP (-) CAT (-), SOD (↓), LP (-/↑)
[72]	CP	21 days (18 h/day)	Wistar, Male	Thyroid	LP	LP (↑)
[45]	MSP	72 h	Wistar, Male	Kidney Erythrocytes	LP	LP (-) LP (↑)
[37]	MSP	4 days	Wistar, Male, 3 months	Plantar muscle Soleus muscle	LP	LP (-) LP (↑)

SD: sleep deprivation; MSP: multiple small platforms; CP: classical platform/inverted flowerpot; Srec: sleep recovery; GSH: Reduced Glutathione; GSSG: Oxidized Glutathione; GPx: Glutathione peroxidase; CAT: Catalase; SOD: Superoxide dismutase; NOx: Nitric oxide; eNOS: Endothelial Nitric Oxide Synthase; p-eNOS: phosphorylated Endothelial Nitric Oxide Synthase; LP: Lipid peroxidation; “↑/↓”: significantly increased/decreased; “-”: not significantly increased or decreased.

**Table 5:** Total sleep deprivation in brain areas.

Reference	SD Protocol	SD Duration	Rat Breed, Sex, Age	Anatomical Site	Oxidative Stress Markers	Results
[29]	GH	6 h	Wistar, Male, 10 weeks	Hippocampus	LP	LP (-)
[42]	GH	12 h	Wistar, Female, 13–15 months	Hypothalamus	nNOS	nNOS (↓)
[73]	ASD	14 days	Wistar, Male	Cortex and Hippocampus	GSH, CAT, SOD, LP	GSH (↓), CAT (↓), SOD (↓), LP (↑)
[74]	DOW	5 days SD + 2 days Srec (3 total cycles) + 3 months Srec	Wistar, Male, Weanling	Hippocampus	GPx, CAT, SOD	GPx (↓), CAT (↓), SOD (↓/-)

SD: sleep deprivation; Srec: sleep recovery; GH: Gentle handling; ASD: Automated sleep deprivation; DOW: Disk over water; GSH: Reduced Glutathione; GPx: Glutathione peroxidase; CAT: Catalase; SOD: Superoxide dismutase; nNOS: neuronal Nitric Oxide Synthase; LP: lipid peroxidation; “↑/↓”: significantly increased/decreased; “-”: not significantly increased or decreased.

**Table 6:** Total sleep deprivation in non-brain areas.

Reference	SD Protocol	SD Duration	Rat Breed, Sex, Age	Anatomical Site	Oxidative Stress Markers	Results
[75]	DOW	5 days	Wistar, Male	Liver	GPx, CAT, SOD, LP	GPx (↓), CAT (↓), SOD (↓), LP (↑)
[76]	DOW	5 days SD + 2 days Srec (3 total cycles)	Wistar, Male, Adult	Liver	GPx, CAT, SOD, LP	GPx (↓), CAT (↓), SOD (↓), LP (↑)
[77]	ASD	14 days 6 h/day–1st week 8 h/day–2nd week	Sprague Dawley, Male, PND 19	Plasma	LP	PND 33: LP (↑) PND 90: LP (-)

SD: sleep deprivation; Srec: sleep recovery; ASD: Automated sleep deprivation; DOW: Disk over water; PND: Postnatal day; GPx: Glutathione peroxidase; CAT: Catalase; SOD: Superoxide dismutase; LP: Lipid peroxidation; “↑/↓”: significantly increased/decreased; “-”: not significantly increased or decreased.

**Table 7:** Gene expression in paradoxical and total sleep deprivation.

Reference	SD Protocol	SD Duration	Rat Breed, Sex, Age	Anatomical Site	Oxidative Stress Measurements	Results
[78]	PSD–MSP	96 h 21 days (18 h/day)	Wistar-Hannover, Male, Adult	Testes	iNOS, eNOS	iNOS (↑), eNOS (↓) iNOS (↑), eNOS (-)
[77]	TSD–ASD	14 days 6 h/day–1st week 8 h/day–2nd week	Sprague Dawley, Male, PND 19	Prefrontal cortex	GPx, CAT, SOD	PND 33: GPx (↑), CAT (-), SOD (↑) PND 90: GPx (-), CAT (-), SOD (-)

SD: sleep deprivation; PSD: paradoxical sleep deprivation; TSD: total sleep deprivation; MSP: multiple small platforms; ASD: automated sleep deprivation; PND: postnatal day; GPx: Glutathione peroxidase; CAT: Catalase; SOD: Superoxide dismutase; eNOS: Endothelial Nitric Oxide Synthase; iNOS: Inducible nitric oxide synthase; “↑/↓”: significantly increased/decreased; “-”: not significantly increased or decreased.

**Table 8:** Serum cortisol/corticosterone changes in sleep deprivation.

Reference	SD Type	SD Protocol	SD Duration	Rat Breed, Sex, Age	Cortisol/Corticosterone
[42]	TSD	GH	12 h	Wistar, Female, 13–15 months	Yes -
[35]	PSD	MSP	24 h	Long-Evans, Male, Old	Yes ↑
[36]	PSD	MSP	72 h	Sprague Dawley, Adult	Yes ↑
[37]	PSD	MSP	4 days	Wistar, Male, 3 months	Yes ↑
[38]	PSD	MSP	5 days	Sprague Dawley, Male, 48 weeks	Yes ↑
[39]	PSD	MSP	20 h 5 days (20 h/day) 5 days (20 h/day) + 5 days Srec	ns	Yes ↑ Yes ↑ Yes -
[40]	PSD	MSP	5 days	Wistar, Male	Yes ↑
[34]	PSD	MSP	14 days (20 h/day)	Sprague Dawley, Male, 12 weeks	Yes ↑
[41]	PSD	MSP	21 days (18 h/day)	Wistar, Male, Peripubertal	Yes ↑
[43]	PSD	MSP	21 days (18 h/day) 21 days (18 h/day) + 5 days Srec 21 days (18 h/day) + 21 days Srec	Sprague Dawley, Male, 12–16 weeks	Yes -

SD: Sleep deprivation; TSD: Total sleep deprivation; PSD: Paradoxical sleep deprivation; Srec: Sleep recovery; GH: Gentle handling; MSP: multiple small platforms; ns: not specified; “↑”: significantly increased; “-”: not significantly increased or decreased.

## 4. Discussion

### 4.1. Sleep Deprivation Determines Changes in Oxidative Stress Parameters

With a few exceptions [26,27,28,29,30,31], most studies included in our review revealed changes in at least one parameter associated with oxidative stress in both PSD and TSD protocols. Even when taking only lipid peroxidation into consideration, with some exceptions [26,27,28,29,31,44,53,54,55,57,58], most studies that determined this parameter revealed elevated levels of lipid peroxidation in both PSD and TSD protocols. Moreover, while some of the results were not interpreted in the context of oxidative stress, most of the observed alterations in these parameters were interpreted as or implied to be evidence of oxidative stress by the original authors. The absence of a specific protocol to selectively eliminate short-wave sleep (SWS) limits our ability to determine its potential antioxidant role based on these results. However, these findings provide additional evidence supporting the antioxidant role of paradoxical sleep and sleep as a whole. 

As previously observed [9], the evidence from the included studies seems to point to changes in oxidative stress parameters in both brain (whole brain, hippocampus, cortex, thalamus, hypothalamus, amygdala, locus coeruleus) and non-brain areas (serum, testes, epididymis, liver, pancreas, aorta, submandibular glands, thyroid, erythrocytes, soleus muscle). However, some body sites did not show changes in oxidative stress parameters: cerebellum and brainstem [26], saliva [66], kidney [45], and plantar muscle [37]. While the evidence is limited in some cases, these observations might suggest that the antioxidant effects of sleep extend beyond the central nervous system, possibly including most body sites. 

Although alterations in oxidative stress parameters have been noted across various body sites, not all sites might exhibit equal resilience to oxidative stress. Interestingly, short SD durations (24–72 h or 48–72 h) might not induce reliable oxidative changes in the hippocampus [26,27,31,44,45,46,47] or cortex [26,28,45,47], as opposed to longer SD protocols. In contrast, changes in oxidative stress parameters seem to be reliably encountered in serum in both short and longer SD durations.

The included studies used multiple rat strains, namely Wistar (39), Sprague Dawley (12), Long-Evans (1), and Wistar-Hannover (1). Given that the majority of these studies employed Wistar rats, strain differences might not be readily apparent. While strain differences might exist and account for some degree of variability in the oxidative stress response to SD [9], the current data seem to indicate the presence of an oxidative stress response after SD across all mentioned rat strains and in both males and females in the case of Wistar [42] and Sprague Dawley rats [49,62].

It has been previously proposed that short SD durations might induce an antioxidant compensatory response in multiple body sites [9,11], evidenced by increases in antioxidants or decreases in oxidants. Contrary to these findings, we did not observe this effect in the studies included in our review that employed short (6–24 h) SD protocols [29,31,35,39,42,64]. Based on these data, we cannot exclude the presence of some compensatory mechanisms in short SD protocols. However, even if such mechanisms are present, their overall effect might not be sufficient to counterbalance the global redox state in the examined organs [79].

### 4.2. Sleep Recovery

Sleep recovery plays a significant role in reversing or at least partially mitigating the effects caused by SD. Somewhat simplified, the sleep recovery time seems to be dependent on the extent of the damage caused by the previous period of SD [43].

Some of the studies included in our review contained sleep recovery periods at the end of the SD period in both PSD [27,33,39,43,70] and TSD [74,76,77] protocols of varying lengths. The presented results are mostly conflicting. While some studies revealed normalizations in multiple parameters related to oxidative stress [39,70], others reported partial recoveries depending on the examined parameter [33] or on the length of the recovery period [43,77]. Lasting changes after sleep recovery were also reported [74,76], as well as the absence of changes after sleep recovery [27]. 

A comparison can somewhat be drawn between the studies that revealed normalizations in oxidative stress parameters [39,70] and studies that reported lasting changes after SD [74,76]. While the available evidence is limited, the results seem to suggest that longer SD periods would induce longer-lasting changes as compared to shorter SD periods. This interpretation would be consistent with the idea that longer SD periods induce increased amounts of ROS and subsequently increased cellular and tissue damage. While age might play a role in the observed results [74], it may be the case that the examined body sites present different sleep recovery dynamics.

Several studies included multiple sleep recovery periods. In one study, a comparison was made between a 5-day sleep recovery period and a longer duration of 21 days, indicating that only the longer recovery period was effective in normalizing the levels of MDA and significantly reducing the ratio of GSH/GSSG compared to the sleep-deprived group [43]. Similarly, another study showed that 57 days of sleep recovery are able to normalize lipid peroxidation and GPx, SOD gene expression in the plasma/prefrontal cortex compared to 24 h of sleep recovery [77]. 

An interesting comparison between animals of different ages is presented by Hernández Santiago et al. [33], suggesting that recovery after SD varies dependent on the age, examined body site and oxidative stress parameter, with differential effects in the liver/pancreas of rats of different ages. Overall, adult rats appear to be more vulnerable to the effects of SD when compared to younger rats. Although the analysis did not find any other studies that investigated the influence of age on sleep recovery dynamics through direct comparisons between animals of different ages, two other studies specifically focused on weanling rats. These studies reported conflicting results: one study revealed a normalization in lipid peroxidation and gene expression of GPx, SOD after 57 days of sleep recovery in plasma/prefrontal cortex [77], while the other reported lasting changes in multiple parameters in the hippocampus following SD and sleep recovery [74].

Sleep recovery periods have also been included in some studies during the experimental protocols in the form of chronic sleep restriction. A consistent pattern seems to emerge from a subset of studies involving daily short-term SD over an extended period of at least two weeks. Several studies published by the same research group indicate that 8 h of daily SD conducted for 4–8 weeks can induce alterations in antioxidant enzymes [51,52,53,54,55,56,57,58,59] but not in lipid peroxidation [53,54,55,57,58] in the hippocampus of adult rats. Contrary to these results, increases in plasma lipid peroxidation seem to be pointed out in the case of young rats, possibly suggesting that early life SD might present a greater oxidative challenge [77]. Conversely, longer daily SD periods (18–20 h/day) for prolonged periods of 14–21 days seem to consistently determine increases in lipid peroxidation in multiple body sites such as the hippocampus [43], testes [34,41], epididymis [32], liver [68] and thyroid [72]. Other findings provide additional support for this trend, as they demonstrate that only the extended SD interval of 22 h per day resulted in alterations in lipid peroxidation, in contrast to the 18 h daily interval [69]. We hypothesize that the reduced daily SD interval may at least partially mitigate the impact of SD on oxidative stress parameters, as evidenced by the absence of lipid peroxidation changes. Furthermore, the absence of increased lipid peroxidation could be attributed to the combined effect of multiple compensatory mechanisms, such as the thioredoxin system and nitric-oxide-mediated inhibition of oxidant enzymes [54].

Conclusions are difficult to draw based on these data. While recovery after an oxidative injury in the context of SD is possible, multiple aspects regarding the dynamics of this process remain unknown. The available data seem to suggest that the process of sleep recovery could potentially impact oxidative stress parameters in a manner that varies based on several factors, including but not limited to the specific organ involved, the examined parameter, the type and duration of SD experienced, animal strain and age, as well as the duration of the subsequent sleep recovery period. Even though they are based on limited data, these findings seem to further suggest that extended periods of SD might induce longer-lasting changes. Furthermore, it appears that longer recovery periods exhibit greater effectiveness in mitigating the observed changes in oxidative stress parameters during SD. In addition, short daily SD intervals seem to be at least partially compensated even if performed for longer time periods. Even though oxidative stress might be transient after SD it has the potential to trigger multiple inflammatory pathways, with downstream effects on synapse formation and neuronal circuit development. These changes may be more evident in younger animals, and the effects could be expressed later in life as anxiety-like and depressive-like behavior [77].

### 4.3. Influence of Sex in Response to Sleep Deprivation

Based on a recent review, available evidence from animal and human studies seem to point out the existence of several nuanced variations in the response to oxidative stress and inflammation between sexes. These differences are likely influenced by the antioxidant properties of sex hormones (mainly estrogens). Specifically, males seem to exhibit greater ROS production, less efficient antioxidant mechanisms, elevated basal inflammation and a comparatively weaker inflammatory response against an acute stimulus [80]. 

Despite the fact that some knowledge regarding variances in sleep and responses to SD between the sexes exists in the literature, there is currently a relative scarcity of comprehensive studies addressing this specific subject matter. Based on the currently limited evidence from animal and human studies, it appears that sleep loss may have a potentially greater impact on cognition in females compared to males [81]. Additionally, some data seem to suggest that females may exhibit a greater stress response to sleep disturbances (HPA reactivity, sympathetic nervous system activation, cardiovascular dysfunction), increased mood disruptions and possibly even an increased inflammatory response [25]. As previously reviewed [82], sex differences between male and female rodents seem to influence multiple characteristics of sleep, such as duration, time spent in different sleep phases, sleep fragmentation, circadian functions and the response to SD. 

Most of the studies included in our review used male animals, with three notable exceptions, which employed mixed male and female groups [49,62] or only female groups [42]. Changes in oxidative stress parameters were observed in all three studies, with similar results in the whole brain and hippocampus [49,62]—decreased antioxidant enzymes and increased lipid peroxidation. Interestingly, Bajaj et al. [42] reported decreases in nNOS in the hippocampus coupled with increases in several inflammatory cytokines, suggesting an influence on sleep–wake cycles determined by SD. 

None of the included studies presented a direct comparison between male and female animals. Changes in oxidative stress parameters have been observed after SD in other studies that employed male and female rats [83,84]. However, to the best of our knowledge, no direct comparison between male and female rats is available in the literature regarding the oxidative stress response in the context of SD.

Considering the established existence of sex-specific variations in the response to oxidative stress and various sleep parameters, it is reasonable to hypothesize that similar distinctions are likely to exist in the context of oxidative stress induced by SD. However, further studies that employ direct comparisons between male and female rodents are required in order to fully characterize this relationship.

### 4.4. Sleep Deprivation and the Stress Response 

Stress and sleep deprivation present an intricate and interconnected relationship whereby SD can elicit a stress response (either directly or indirectly), and in turn, stress can determine multiple effects on sleep. The activation of the HPA axis serves as one of the central mechanisms through which the interplay between stress, sleep/sleep deprivation and metabolism is played out [85].

The assessment of the HPA axis activation and associated stress response typically involves quantifying serum cortisol levels in humans and corticosterone levels in rodents. Although corticosterone is the primary glucocorticoid in rodents, it has been shown that both cortisol and corticosterone exhibit similar dynamics (though not identical) and can be measured in order to assess the response to a stressor [86]. 

The significance of the stress response during SD stems from its potential to induce oxidative stress. This connection poses challenges in the interpretation of the effects attributed to SD. Various forms of stress, accompanied by elevated levels of stress hormones, are known for their ability to induce oxidative stress changes in multiple body sites on rat models such as the brain [87,88,89], liver [87,89,90], kidney [87,89], heart, stomach [87] and testes [91,92]. 

Most of the studies included in our review that measured serum cortisol/corticosterone revealed increases in these parameters, at different SD durations, during both continuous SD and sleep restriction protocols. Of note, most of these studies employed PSD protocols, and only one study used a TSD protocol [42]. Only two of the included studies reported insignificant changes in the levels of serum cortisol/corticosterone [42,43]. Bajaj et al. [42] observed increases in serum cortisol in the sleep-deprived group, although these changes did not reach statistical significance. This fact can be at least partially explained by the short SD duration of 12 h, exclusive inclusion of female animal groups or the gentle handling protocol used. However, it is worth noting that the results presented by Konakanchi et al. [43] are in contradiction to those reported by other authors.

Given the fact that only one of the included studies employed a TSD protocol with a short SD duration [42], we believe that a comparison between the stress-inducing effects of PSD and TSD protocols is not possible based on these findings.

Only two of the included studies evaluated the effects of sleep recovery at the end of the SD period on stress hormones [39,43]. Even though limited, the results seem to indicate that 5 days of sleep recovery are sufficient to normalize serum corticosterone levels after 5 days of SD [39]. Based on previous evidence, it appears that the normalization of the increased corticosterone/ACTH levels induced by SD might occur at an even faster rate after 24 h [93] or even after 4 h [94], depending on the experimental conditions such as the length of the previous sleep deprivation paradigm. Nevertheless, SD may potentially still present longer-lasting effects on multiple hormones and neurotransmitters [93], including the stress response, by influencing the reaction to a subsequent stressor [94,95].

It is currently believed that the stress response observed in rodent models following SD is likely determined by a combination of stressors originating both directly from SD and those induced by the SD protocol itself. While the extent and differences between SD protocols in inducing stress are controversial, multiple studies seem to suggest that the most commonly used SD protocols induce a variable stress response in rodents [96,97,98]. In certain SD protocols, such as the MSP or CP paradigms, it is possible to at least partially assess the source and possible influence of the stress response by introducing a second control group placed on wide platforms or a steel grid that allow for undisturbed sleep. Although the utilization of a large platform group is known to have certain limitations, such as disrupted normal sleep patterns [99] and even a potential stress response [96], it provides additional insights for understanding the stress response during SD. In our review, less than half of the studies that used the MSP or CP paradigms employed the use of a second wide platform/grid control group (40.4%, *n* = 19/47) [26,36,38,43,48,51,52,53,54,55,56,57,58,59,60,61,69,70,72]. Interestingly, with the exception of one study [60], all studies consistently indicated the absence of significant differences in the assessed oxidative stress parameters between the cage control and the wide platform/grid control group. Furthermore, in three of the aforementioned studies, serum cortisol/corticosterone levels were also assessed, indicating no statistically significant differences between the control groups [36,38,43]. 

Overall, the presence of a stress response after SD is well-known (see reviews [11,98]). Despite the elevated levels of stress hormones observed in the majority of studies included in the analysis, the precise underlying cause of this response still remains somewhat unclear. Previous research suggests that stress determined by SD may exhibit distinct characteristics when compared to other forms of stress. These include a gradual rise in corticosterone, a lack of habituation in the HPA axis, a diminished response in ACTH and the absence of increases in adrenal gland size [94]. 

Most of the results presented in our review indicate elevated corticosterone levels after SD, coupled with a lack of notable differences between multiple control groups in oxidative stress parameters and stress hormones. While limited to the presented data, these results might suggest that sleep deprivation may itself act as the primary determinant of the observed stress response rather than the specific experimental protocol. If this is the case, the observed changes in oxidative stress parameters are most likely a direct consequence of sleep deprivation and not determined by the stress of the experimental protocol. 

To further complicate matters, stress might play a physiological role in the induction of sleep, potentially facilitating the occurrence of sleep rebound subsequent to exposure to a stressor. This process is presumed to play an evolutionary role in mitigating the effects associated with stress exposure [100]. Furthermore, the stress response determined by SD may be physiological in nature, playing a crucial role in facilitating REM sleep rebound after SD [101]. 

The timing of measurement represents another crucial aspect to consider when examining the stress response during SD. As previously reviewed [98], the measurement of stress hormones at the end of the SD protocol may introduce a potential source of bias, as it could mask transient increases in stress hormone levels that occur at the beginning of the procedure.

### 4.5. Mechanisms of Sleep Deprivation-Induced Oxidative Stress

The literature on experimental SD frequently exhibits ambiguity, as multiple studies demonstrate the absence of oxidative stress following sleep deprivation [26,27,28,29,30,31,102,103]. This fact may likely be attributed primarily to the overall variability in experimental conditions. However, most evidence from a previous systematic review [9] and from the current review seems to point towards the fact that sleep deprivation represents an oxidative challenge.

The precise mechanism through which sleep deprivation induces oxidative stress remains incompletely understood. However, from a conceptual standpoint, heightened levels of ROS could arise as a result of increased production, reduced clearance, or a combination of both mechanisms. Moreover, sleep deprivation might give rise to distinct adverse conditions that determine the accumulation of ROS [104]. If oxidative stress is indeed an outcome of sleep deprivation, it is likely attributable to a complex interplay of various site- and possibly species-specific mechanisms, including but not limited to: increased glucose consumption during prolonged waking, stress hormones induced oxidative stress, increased metabolism and mitochondrial dysfunction, endoplasmic reticulum dysfunction, and gut dysbiosis linked to Nox enzyme (NADPH oxidase) induced ROS hyperproduction in the gut. 

Physiological sleep is distinguished by a notable reduction in whole-body energy expenditure, with slow-wave sleep, in particular, exhibiting a decrease of approximately 15–35% [105]. Moreover, the diminished neuron activity and metabolism observed during sleep contribute to a reduction in brain glucose utilization [105,106,107]. As a result, sleep deprivation and prolonged wakefulness might lead to heightened metabolism, increased glucose consumption and increased production of ROS [43,104].

As previously shown, the activation of the HPA axis and the consequent release of stress hormones may induce oxidative stress in various body sites, including the central nervous system and the periphery [87,88,89,90,91,92]. Regardless of its specific origin, the observed stress response during sleep deprivation may, to some extent, contribute to the generation of ROS in multiple body sites.

As previously reviewed, mitochondrial dysfunction, endoplasmic reticulum dysfunction and gut dysbiosis might be other mechanisms through which SD generates oxidative stress [104]. The heightened metabolism observed during SD translates into increased mitochondrial oxygen-dependent ATP synthesis, consequently leading to an elevated production of ROS. The accumulation of misfolded or unfolded proteins observed during SD can induce oxidative stress by imposing an increased demand for protein folding processes. Moreover, the observed gut dysbiosis during SD could potentially contribute to the generation of ROS by triggering the hyperactivation of the Nox enzyme, which is responsible for a significant portion of ROS production in the gut.

Additionally, certain data suggest a connection between insufficient sleep duration and heightened susceptibility to acute oxidative stress. This phenomenon could potentially be attributed to an elevated baseline accumulation of ROS, leading to an increased sensitization to acute oxidative stress [10].

### 4.6. Negative Health Outcomes Associated with Sleep Deprivation

There are still several unresolved questions concerning the relationship between SD and various pathologies. SD has been linked to a diverse array of both acute and chronic adverse health consequences, encompassing daytime sleepiness, mental health disorders, neurodegenerative disorders, metabolic disorders such as diabetes and obesity, as well as cardiovascular disease (CV), among other conditions [2,3,4,108].

From a conceptual standpoint, SD can be viewed as a stressor that induces an increase in the allostatic load, thereby contributing to dysfunctions across multiple organ systems. The increase in allostatic load is likely mediated through the multiple consequences of SD and circadian disruption, such as oxidative stress, inflammation and multiple hormone dysregulations (Cortisol, Insulin, etc.) [11].

Multiple mechanistic links have been proposed in order to explain the previously mentioned SD-induced pathologies. In the CV system, SD is reported to induce endothelial dysfunction (through chronobiological disruption, oxidative stress, inflammation, and autonomic dysregulation) [108] and hypertension [109,110]. Furthermore, short sleep durations have been associated with an increased risk of metabolic syndrome [111], obesity and diabetes [112], further increasing CV risk factors. Multiple mechanisms have been proposed to explain the effects of sleep deprivation on metabolism, such as changes in energy intake (increased hunger and appetite) and possibly expenditure, hormonal changes (decreased Leptin and glucagon-like peptide-1, increased Ghrelin, altered Cortisol rhythms), inflammation and oxidative stress, sympathetic predominance (either from reduced vagal tone or increased sympathetic activity). Overall, these mechanisms may lead to Insulin resistance and altered energy metabolism (reviewed in [112]). Previous studies have shown that SD leads to an elevated food intake in both human subjects and rodent models. However, it is noteworthy that differential effects on overall weight have been observed, with rodents experiencing weight loss and humans exhibiting weight gain [113]. SD has been shown to affect various biochemical and molecular pathways found in multiple neurological or neurodegenerative disorders such as Alzheimer’s disease (AD), Parkinson’s disease, Multiple sclerosis, Huntington’s disease and stroke [10,114]. Overall, the proposed main mechanisms through which SD determines these changes might be summarised as: decreased neuronal clearance of misfolded proteins (α-synuclein, amyloid-β, tau), impairment of the glymphatic system, neuroinflammation and oxidative stress (reviewed in [114]). Furthermore, it is important to note that oxidative stress can induce protein misfolding and aggregation, genomic instability and DNA damage. Consequently, the neuronal accumulation of ROS might be seen as a contributing element in the pathogenesis of neurodegenerative diseases [10,33]. SD has also been shown to determine the activation of astrocytes and microglia in the central nervous system, leading to neuronal injury through increased levels of proinflammatory factors [115]. SD determines alterations in multiple pathways that may lead to the pathology observed in AD, such as neuroinflammation and oxidative stress, endothelial damage, impaired glial pathway, inhibition of neurogenesis and cholinergic neurons, impairment of spatial and working memory, impairment of long-term potentiation and synaptic plasticity, decreased amyloid-β clearance and subsequent accumulation of amyloid-β and tau [114]. In addition, SD has been shown to directly negatively influence memory. While the exact mechanisms through which SD determines memory impairment are not completely known, multiple pathways have been proposed, such as oxidative stress, neuroinflammation, neuronal damage, decreased synaptic plasticity, changes in neurotransmitters and gene expression (reviewed in [116]).

## 5. Limitations

Our review presents several limitations determined by the design of the study, by the original studies from which the data were extracted and by the existing experimental sleep deprivation protocols.

### 5.1. Limitations Inherent to the Review Methodology and Results

The first limitation of our review refers to the search algorithm. While we employed systematic search strategies, the searches were performed in three major indexing databases and included studies published between 2015 and 2022. Studies published before 2015 were not included in order to avoid duplications with the previous review written by Villafuerte et al. [9]. Most of the included studies utilized PSD protocols through the MSP paradigm. All included studies utilized a limited number of rat strains as the SD model, with Wistar rats being the most frequently used. As a consequence of these factors, the findings presented in our review cannot be widely generalised to other rodent models, such as mice. Even though strain differences are not evident from these data, they have been suggested before in the context of SD and may account for at least some degree of variability in the observed results [9]. 

### 5.2. Limitations Inherent to the Original Studies

We encountered several challenges associated with data extraction due to the often limited and unclear aspects regarding the methodology of the included studies. These include the general timeline of the experiments (mainly the presence of sleep recovery periods after SD and until euthanasia), stress reduction procedures such as maintaining social hierarchy in the case of the MSP paradigm or limited human interaction and the description of the control group. 

The majority of the studies included in the analysis utilized behavioral tests. However, it was not consistently indicated whether these tests were conducted during or at the end of the SD protocol. To address this potential bias, sleep recovery periods were taken into consideration only when explicitly described as such by the original studies or when presented on a clear experimental timeline.

Furthermore, we observed an increased variability in the implementation of the specific SD protocols among the included studies. 

### 5.3. Limitations Determined by the Existing Sleep Deprivation Protocols

The studies included in our review employed a multitude of sleep deprivation protocols, such as MSP, CP, DOW, GH and ASD. Sleep deprivation research has been carried out since the 1950s, leading to the development of multiple protocols aimed at eliminating sleep. An ideal SD protocol would be able to completely abolish a desired sleep phase and would be free from any inherent bias factors associated with the procedure itself. Additionally, such a protocol should include a comparable control group not exposed to SD. It is well-known that all the currently available SD protocols suffer from some drawbacks [9,117]. Previous research has shown that the most commonly used PSD protocols could effectively eliminate or nearly eliminate REM sleep [99,118]. However, while some data seem to point out an additional partial reduction in SWS (37% SWS reduction in socially stable rats in the case of MSP and 31% SWS reduction in the case of CP) [99], other studies have reported minimal reductions in SWS through the CP paradigm [118]. Furthermore, as stated before, most SD protocols might be stress-inducing [96,97,98]. 

## 6. Quality and Bias Evaluation of Included Studies

A limited quality and bias evaluation was performed for all included studies by examining the following criteria: control group description, randomisation, total number of experimental animals and social hierarchy maintenance in the case of studies that employed the MSP paradigm. Such an evaluation was mainly motivated by the frequent variations in the employed SD protocols. Overall, the studies included in our analysis were of good methodological quality. Consequently, no studies were excluded based on this criterion.

All included studies contained at least one control group that was not exposed to sleep deprivation. More than two-thirds of the included studies (72.2%, *n* = 39/54) presented a detailed description of the control group, while the remaining studies specified the existence of a control group without providing a detailed description. Moreover, some studies that employed the MSP or CP paradigm included two separate control groups: cage control and wide platform/grid control (40.4%, *n* = 19/47).

More than half of the included studies specified the use of randomisation in the creation of the groups (66.6%, *n* = 36/54), but only one study offered a detailed explanation of the randomisation method. 

In the majority of the included studies, the total count of experimental animals was explicitly provided or could be inferred from the sample sizes (77.7%, *n* = 42/54), whereas in a limited number of studies, it remained unclear (22.2%, *n* = 12/54).

The evaluation of social hierarchy maintenance was motivated by two factors: the frequent use of the MSP SD protocol in the included studies and the effectiveness of this procedure to further mitigate stress within the MSP paradigm [119]. Of the included studies that employed the MSP sleep deprivation protocol, only a minority (13.5%, *n* = 5/37) provided, at the very least, partial insights regarding the maintenance of social hierarchy throughout the experimental period. The remaining studies either lacked any specific information regarding the maintenance of social hierarchy or provided unclear data. 

## 7. Conclusions

The inherent variability in sleep deprivation protocols coupled with factors such as possible sex and differences frequently adds a layer of complexity to the interpretation of data regarding sleep deprivation. As such, definitive conclusions are difficult to draw regarding the multifaceted relationship between sleep deprivation and oxidative stress. 

The currently available data seem to further suggest that both paradoxical and total sleep deprivation can determine alterations in oxidative stress parameters. These changes seem to be relatively consistent and can be seen in both brain (whole brain, hippocampus, cortex, thalamus, hypothalamus, amygdala, locus coeruleus) and non-brain areas (serum, testes, epididymis, liver, pancreas, aorta, submandibular glands, thyroid, erythrocytes, soleus muscle), in multiple rat strains (Wistar, Sprague Dawley, Long-Evans, Wistar-Hannover) and in both males and females in the case of Wistar and Sprague Dawley rats.

Sleep recovery seems to be characterized by extensive variability determined by a multitude of factors ranging from the duration of sleep deprivation to the duration of sleep recovery, among others. Furthermore, short daily sleep deprivation seems to be at least partially compensated even when performed for longer time periods, at least when considering lipid peroxidation. 

Most available data seem to suggest the presence of a stress response after sleep deprivation. Stress has traditionally been considered a significant confounding variable in studies investigating sleep deprivation. However, the origin of the stress response and the overall effects of stress in SD studies remain somewhat unclear. The findings outlined in this review seem to provide evidence that supports the hypothesis of sleep deprivation being a stressor in itself. Consequently, the oxidative changes observed during sleep deprivation are most likely a direct result of sleep deprivation rather than an indirect effect of the experimental conditions.

## 8. Future Directions

Multiple unknowns remain regarding the relationship between oxidative stress and sleep deprivation, requiring to be addressed in future studies: sex differences in response to sleep deprivation and sleep recovery, dynamics of sleep recovery, the existence of an antioxidant compensatory mechanism in short SD durations and the molecular mechanisms through which SD determines oxidative stress. 

## Data Availability

All data are contained within the article and Supplementary file.

## Supplementary Materials

The following supporting information can be downloaded at: https://www.mdpi.com/article/10.3390/antiox12081600/s1, Supplementary file S1.
