# Supplementary material for: Sleep Deprivation-Induced Oxidative Stress in Rat Models: A Scoping Systematic Review
Source: Antioxidants (Basel). 2023 Aug 11;12(8):1600. doi: 10.3390/antiox12081600 (PMC10451248; doi:10.3390/antiox12081600)
Supplement: Supplementary file 1 [file antioxidants-12-01600-s001.zip › antioxidants-2534247-supplementary.pdf]

Sleep Deprivation-Induced Oxidative Stress in Rat Models: A Scoping Systematic Review

Supplementary File S1

The methods used in this review were a priori designed and based on the 22 item Preferred Reporting Items for Systematic Reviews and Meta-Analyses extension for Scoping Reviews (PRISMA - ScR) [1].

1. Search strategy

1.1 Search strategy

The search algorithm was developed by H.A.C. (research methodology, medical information and biostatistics specialist) and performed by V.S.N. and D.A.T. The search algorithm was not previously peer reviewed using the Peer Review of Electronic Search Strategies (PRESS) checklist.

1.1.1 Keywords used during the search

Keywords and MESH terms used in the search algorythm

**Sleep deprivation**	**Oxidative stress**	**Sleep apnoea and other diseases**	**Rat studies**	**Mesh terms**
Sleep	"Oxidative stress"	"Sleep apnea"	Rat	Sleep
"Sleep depriv*"	"Oxidative damage*"	"Sleep apnoea"	Rats	Sleep deprivation
	"Oxidative stress injur*"	"Sleep apnea syndrome"	Rattus	Oxidative stress
"Total sleep depriv*"	"Lipid peroxidation*"	"Sleep apnoea syndrome"	"Rattus norvegicus”	Lipid peroxidation
"Partial sleep depriv*"	Glutathione	"Sleep Hypopnea"	"Albino Rat"	Glutathione
"REM sleep depriv*"	"Glutathione peroxidase"	"Intermittent hypoxia"	"Norway Rat"	Glutathione Peroxidase
"Paradoxical sleep depriv*"	GSH	"neurodegenerative disease*"	Wistar	Nitric Oxide
TSD	GSSG	neurodegenerative	"Wistar rat"	Catalase
PSD	GPx		"Long-Evans"	Superoxide dismutase
	"Nitric Oxide"		"Long Evans"	Sleep Apnea Syndromes
	"Nitrogen Monoxide"		"Long-Evans rat"	Neurodegenerative Diseases
	Catalase		"Long Evans rat"	Rats
	CAT		"Sprague-Dawley"	
	"Superoxide dismutase"		"Sprague Dawley"	
	SOD		"Sprague-Dawley rat"	
			"Sprague Dawley rat"	
			Rodent*	

1.1.2 Simplified search strategy

(sleep OR sleep deprivation) AND (oxidative stress) AND (rat) AND (publication date 01.01.2015-01.10.2022) NOT (sleep apnea OR neurodegenerative disease)

1.1.3 Pubmed Search strategy – Results: 152

("Sleep"[MeSH Terms] OR "Sleep Deprivation"[MeSH Terms] OR "Sleep depriv*"[tiab] OR "Total Sleep depriv*"[tiab] OR "Partial sleep depriv*"[tiab] OR "REM sleep depriv*"[tiab] OR "Paradoxical sleep depriv*"[tiab] OR "TSD"[tiab] OR "PSD"[tiab]) AND ("Oxidative Stress"[Mesh] OR "Lipid Peroxidation"[Mesh] OR "Glutathione"[Mesh] OR "Glutathione Peroxidase"[Mesh] OR "Nitric Oxide"[Mesh] OR "Catalase"[Mesh] OR "Superoxide Dismutase"[Mesh] OR "Oxidative Stress"[tiab] OR "Oxidative damage*"[tiab] OR "Oxidative stress injur*"[tiab] OR "Lipid Peroxidation"[tiab] OR "Glutathione"[tiab] OR "Glutathione peroxidase"[tiab] OR "GSH"[tiab] OR "GSSG"[tiab] OR "GPx"[tiab] OR "Nitric Oxide"[tiab] OR "Nitrogen monoxide"[tiab] OR "Catalase"[tiab] OR "CAT"[tiab] OR "Superoxide Dismutase"[tiab] OR "SOD"[tiab]) AND ("Rats"[Mesh] OR "rat"[tiab] OR "rats"[tiab] OR "rattus"[tiab] OR "Rattus norvegicus"[tiab] OR "albino rat"[tiab] OR "Norway Rat"[tiab] OR "wistar"[tiab] OR "wistar rat"[tiab] OR "long-evans"[tiab] OR "long evans"[tiab] OR "long-evans rat"[tiab] OR "long evans rat"[tiab] OR "sprague-dawley"[tiab] OR "sprague dawley"[tiab] OR "sprague-dawley rat"[tiab] OR "sprague dawley rat"[tiab] OR "rodent*"[tiab]) AND (2015/1/1:2022/10/01[dp]) NOT ("Sleep Apnea Syndromes"[Mesh] OR "Neurodegenerative Diseases"[Mesh] OR "sleep apnea"[tiab] OR "sleep apnoea"[tiab] OR "sleep apnea syndrome*"[tiab] OR "sleep apnoea syndrome*"[tiab] OR "sleep hypopnea"[tiab] OR "intermittent hypoxia"[tiab] OR "neurodegenerative disease*"[tiab] OR "neurodegenerative"[tiab])

1.1.4 Web of Science search strategy – Results: 398

TS=("Sleep" OR "Sleep deprivation" OR "sleep depriv*" OR "Total Sleep depriv*" OR "Partial sleep depriv*" OR "REM sleep depriv*" OR "Paradoxical sleep depriv*" OR "TSD" OR "PSD") AND TS=("Oxidative stress" OR "Lipid peroxidation" OR "Glutathione" OR "Glutathione peroxidase" OR "Nitric oxide" OR "Catalase" OR "Superoxide Dismutase" OR "Oxidative damage*" OR "oxidative stress injur*" OR "GSH" OR "GSSG" OR "GPx" OR "Nitrogen monoxide" OR "CAT" OR "SOD") AND TS=("rats" OR "rat" OR "rattus" OR "rattus norvegicus" OR "albino rat" OR "norway rat" OR "Wistar" OR "Wistar rat" OR "Long-Evans" OR "Long Evans" OR "Long-Evans rat" OR "Long evans rat" OR "Sprague-Dawley" OR "Sprague Dawley" OR "sprague-dawley rat" OR "sprague dawley rat" OR "rodent*") AND DOP=(2015-01-01/2022-10-01) NOT TS=("sleep apnea syndrome*" OR "sleep apnea" OR "sleep apnoea" OR "sleep apnoea syndrome*" OR "sleep hypopnea" OR "intermittent hypoxia" OR "neurodegenerative disease*" OR "neurodegenerative")

1.1.5 Cochrane library – Results: 4

ID Search Hits

#1 MeSH descriptor: [Sleep] explode all trees 7258

#2 MeSH descriptor: [Sleep Deprivation] explode all trees 932

#3 ("sleep depriv*" OR "Total Sleep depriv*" OR "Partial sleep depriv*" OR "REM sleep depriv*" OR "Paradoxical sleep depriv*" OR "TSD" OR "PSD") 828

#4 #1 OR #2 OR #3 8008

#5 MeSH descriptor: [Oxidative Stress] explode all trees 3785

#6 MeSH descriptor: [Lipid Peroxidation] explode all trees 1094

#7 MeSH descriptor: [Glutathione] explode all trees 758

#8 MeSH descriptor: [Glutathione Peroxidase] explode all trees 500

#9 MeSH descriptor: [Nitric Oxide] explode all trees 2470

#10 MeSH descriptor: [Catalase] explode all trees 313

#11 MeSH descriptor: [Superoxide Dismutase] explode all trees 924

#12 ("Oxidative stress" OR "Lipid peroxidation" OR "Glutathione" OR "Glutathione peroxidase" OR "Nitric oxide" OR "Catalase" OR "Superoxide Dismutase" OR "Oxidative damage*" OR "oxidative stress injur*" OR "GSH" OR "GSSG" OR "GPx" OR "Nitrogen monoxide" OR "CAT" OR "SOD") 30052

#13 (#5 OR #6 OR #7 OR #8 OR #9 OR #10 OR #11 OR #12) 30062

#14 MeSH descriptor: [Rats] explode all trees 1445

#15 ("rats" OR "rat" OR "rattus" OR "rattus norvegicus" OR "albino rat" OR "norway rat" OR "Wistar" OR "Wistar rat" OR "Long-Evans" OR "Long Evans" OR "Long-Evans rat" OR "Long evans rat" OR "Sprague-Dawley" OR "Sprague Dawley" OR "sprague-dawley rat" OR "sprague dawley rat" OR "rodent*") 11579

#16 (#14 OR #15) 11579

#17 MeSH descriptor: [Sleep Apnea Syndromes] explode all trees 3377

#18 MeSH descriptor: [Neurodegenerative Diseases] explode all trees 12589

#19 ("sleep apnea syndrome*" OR "sleep apnea" OR "sleep apnoea" OR "sleep apnoea syndrome*" OR "sleep hypopnea" OR "intermittent hypoxia" OR "neurodegenerative disease*" OR "neurodegenerative") 10704

#20 (#17 OR #18 OR #19) 22546

#21 #4 AND #13 AND #16 NOT #20 with Cochrane Library publication date Between Jan 2015 and Oct 2022

1.2 Search date and total results

All searches were performed between 21-23 February 2023. The final searches were performed on 23 February 2023. The searches were saved and exported into Endnote 20 (IBM - Clarivate) for management.

The combined searches yielded a total of 554 results.

1.3 Study inclusion flowchart

The combined searches yielded a total of 554 results (Web of Science = 398, PubMed = 152, Cochrane Library = 4).

A total of 127 duplicated records were identified and removed.

A total of 427 records were screened by reading the title and abstract and a total of 347 records were excluded during this stage.

A total of 80 reports were sought for retrieval and 5 reports were not retrieved due to inaccesibility.

A total of 75 reports were assessed for eligibility by reading the full text and 21 were excluded due to not meeting the inclusion criteria.

Finally, a total of 54 studies were included in the review.

1.4 Inclusion and exclusion criteria

1.4.1 Inclusion criteria

- Experimental sleep deprivation studies performed on rat models, with oxidative stress measurements
- All sleep deprivation protocols were accepted (total sleep deprivation and paradoxical sleep deprivation); Automated sleep deprivation protocols (Forced locomotion/ sensory stimulation etc) were also included. These protocols were excluded only if the original authors specified that the protocol was used for sleep fragmentation
- Studies on rats, irrespective of sex, age, weight, strain
- Publication date: 01.01.2015-01.10.2022
- Language: Only studies written in English were included
- Presence of at least one of the following oxidative stress measurements: GSH, GSSG, GSH/GSSG ratio, CAT, SOD, NO (Nitric oxide, eNOS, iNOS, nNOS), Lipid peroxidation (irrespective of the modality of determination – Enzyme activity, PCR, Western Blot, Immunohistochemistry, Immunofluorescence etc)

1.4.2 Exclusion criteria

- Experimental sleep deprivation studies on humans or other animal models
- Pregnancy with/ without effects on offspring
- Lack of oxidative stress measurements as specified in the inclusion criteria
- Circadian disruption protocols / hypoxia protocols / sleep fragmentation protocols
- Narrative reviews/ systematic reviews/ meta-analyses

1.4.3 Inclusion and exclusion criteria specifications

The decision to incorporate studies published between 2015 and 2022 was prompted by the prior review authored by Villafuerte et al [2] and published in 2015. This measure was taken to prevent any potential duplications within the existing review.

This review was focused on investigating oxidative stress alterations specifically in rat models. It is worth noting that strain differences have been previously documented between mice and rats [2]. However, conducting a comprehensive analysis encompassing all existing animal and human models of sleep deprivation would have exceeded the intended scope of this review.

A broad selection of markers is available to evaluate the occurrence of oxidative stress. However, attempting to extract all existing oxidative stress measurements would have been unfeasible.

Therefore, the aforementioned oxidative stress measurements were specifically chosen based on their recurrent appearance in the included studies during the data charting process.

Studies that employed circadian disruption, hypoxia (obstructive sleep apnoea models) or sleep fragmentation protocols were excluded due to a potential bias in the interpretation of the results.

1.5 Comparisons between groups

Comparisons were sought between a control group without sleep deprivation and a group that was sleep deprived, without any other interventions or drug administration. Although various treatment approaches were employed in most studies to modulate oxidative stress, the extracted data specifically pertains to two distinct groups: the untreated control group and the untreated sleep-deprived group.

Vehicle administration, procedures specific to sleep deprivation protocols (electrode implantation etc) and behavioural tests were not considered as reasons to exclude studies from the current review.

1.6 Additional data

Title and abstract screening, full text evaluation and data extraction were performed by two researchers (V.S.N and I.A.C).

A data charting form was initially piloted and developed by two authors (V.S.N and I.A.C) and updated as needed. Similarly, a data extraction form was further piloted based on five included studies.

All discrepancies were resolved by consensus and oversight from C.C., H.A.C. and S.C.

No automation tools were employed during the search, title and abstract screening, full text evaluation or data extraction steps of the review.

2. Exclusion codes used during abstract and full text screening

2.1 Title and abstract screening exclusion codes

Title and abstract screening exclusion codes

Exclusion code	Code explanation
Not related subject	Studies not related to the main subject of the review – experimental sleep deprivation studies performed on rats, with oxidative stress measurements
Publication type	Other study types than experimental studies (ex. Narrative reviews, systematic reviews, meta-analyses, book chapters etc.)
Other model	Experimental sleep deprivation studies with oxidative stress measurements that employed other models than rats (humans, other rodent or animal models etc)
Language	Studies published in any other language than English
Date	Studies published outside of the interval 01.01.2015-01.10.2022

2.2 Full text assessment exclusion codes

Full text assessment exclusion codes

Exclusion code	Code explanation
No groups for comparison	No suitable groups for comparison. Studies were excluded if a direct comparison between a control group and a sleep deprived group was not available. This includes control groups that were exposed to sleep deprivation or subjected to drug administration other than vehicle.
No measurements	Absence of the following oxidative stress measurements: GSH, GSSG, GPx, CAT, SOD, Nitric oxide (Nitric oxide, iNOS, eNOS, nNOS), Lipid peroxidation
Unclear data	Unclear data regarding the main aspects of the review – sleep deprivation protocol or duration, group formation, oxidative stress measurements
Circadian rhythm	Studies that indicated deviation from a standardized 12-12 hour light/dark schedule were excluded, due to a potential risk of bias determined by circadian rhythm alterations

3. Data extraction

3.1 Extracted data

Data extraction was performed independently by two authors (V.S.N and I.A.C).

A standardised form (intially piloted on five included studies) was used for data extraction. The following data was extracted from each included study: sleep deprivation protocol and duration, type of control group, subject characteristics (rat breed, age and sex), serum stress hormones measurements (cortisol/ corticosterone), oxidative stress measurements (GSH, GSSG, GSH/GSSG ratio, CAT, SOD, Nitric oxide, Lipid peroxidation).

All measurement types for the mentioned parameters were included. Gene expression results were included and presented as separate results.

In the case of Nitric Oxide the following measurements were included: Nitric Oxide, enzymes involved in the Nitric oxide synthesis cycle (eNOS – endothelial Nitric oxide synthetase, iNOS – inductible Nitric oxide synthetase, nNOS – neuronal Nitric oxide synthetase), irrespective of the measurement modality.

In the case of Lipid peroxidation all measurement types were included: Malonaldehyde (MDA), Thiobarbituric acid reactive substances (TBARS), 8-isoprostane etc.

3.2 Age of subjects in the original studies

The age of the subjects was extracted according to the methodology outlined in the original research. The age at the beginning of the experiments was prioritised over the age of acquisition only in cases in which the original authors provided a clear timeline of the experiments.

3.3 Sleep deprivation protocols

The sleep deprivation protocols were extracted according to the specifications provided in the original studies. In certain instances, priority was given to the detailed description of the protocol rather than just the mentioned type of protocol.

4. Results

The full-length Results are presented in Tables S1-S7 for paradoxical sleep deprivation and total sleep deprivation in both brain and non-brain areas. These Tables represent the full-length version of the Tables 1-7 from the main text.

The included studies were grouped by type of SD protocol (PSD or TSD), by anatomical region in which the oxidative stress measurements were performed and by the length of the SD duration. In some cases, studies that performed oxidative stress measurements in multiple body sites were presented in multiple tables according to the previously detailed grouping.

Table S1. Paradoxical sleep deprivation in the hippocampus

Reference	SD protocol	SD duration	Rat breed, sex, age	GSH	GSSG	GSH/GSSG ratio	GPx	CAT	SOD	Nitric oxide	Lipid peroxidation
Sahin et al [3]	MSP	48 h	Wistar, Male, PND 28								Yes - (MDA)
Aboul Ezz et al [4]	MSP	48 h	Wistar, Male, Adult	Yes ↓						Yes ↑ (NOx)	Yes - (MDA)
Turan et al [5]	MSP	72 h	Wistar, Male, Adult	Yes -							Yes - (MDA)
Roubalová et al [6]	MSP	72 h	Wistar, Male								Yes ↑ (MDA)
Zuo et al [7]	MSP	72 h	Wistar, Male, Adult	Yes ↓					Yes ↓		Yes ↑ (MDA)
Vosahlikova et al [8]	MSP	72 h	Wistar, Male, Adult								Yes ↑ (MDA)
Nabaee et al [9]	MSP	24, 48, 72 h	Wistar, Male, Adult	Yes -			Yes -	Yes -	Yes -		Yes - (MDA)
Kwon et al [10]	CP	4 days	Wistar, Male, 5 weeks								Yes ↑ (4-HNE) *

Zhang et al [11]	MSP	5 days	Sprague-Dawley, Male and Female, 6 weeks			Yes ↓	Yes -	Yes ↓	Yes ↓		Yes ↑ (MDA)
Gou et al [12]	MSP	5 days	Sprague-Dawley, Male, 48 weeks							Yes ↑ (NOx)	
Andrabi et al [13]	CP	6 days (48h SD, 48h Srec, 48h SD, 48h Srec, 48h SD)	Wistar, Male, Adult					Yes -			Yes ↑ (MDA)
Konakanchi et al [14]	MSP	21 days (18h/ day)<br><br>21 days (18h/ day) + 5 days SRec<br><br>21 days (18h/ day) + 21 days Srec	Sprague-Dawley, Male, 12-16 weeks			Yes ↓<br><br>Yes ↓<br><br>Yes ↓					Yes ↑ (MDA)<br><br>Yes ↑ (MDA)<br><br>Yes - (MDA)
Alzoubi et al [15]	MSP	4 weeks (8h / day)	Wistar, Male, Adult	Yes -	Yes ↑	Yes ↓	Yes ↓	Yes ↓			
Alzoubi et al [16]	MSP	4 weeks (8h / day)	Wistar, Male, Adult	Yes -	Yes ↑	Yes ↓	Yes ↓	Yes ↓			
Alzoubi et al [17]	MSP	4 weeks (8h / day)	Wistar, Male, Adult	Yes -	Yes ↑	Yes ↓	Yes ↓	Yes ↓	Yes ↓		Yes - (TBARS)
Massadeh et al [18]	MSP	6 weeks (8h / day)	Wistar, Male, 8-10 weeks	Yes -	Yes ↑	Yes ↓	Yes ↓	Yes ↓	Yes ↓		Yes - (TBARS)
Alzoubi et al [19]	MSP	6 weeks (8h / day)	Wistar, Male, Adult	Yes -	Yes ↑	Yes ↓	Yes ↓	Yes ↓	Yes ↓		Yes - (TBARS)
Alzoubi et al [20]	MSP	6 weeks (8h / day)	Wistar, Male, Adult	Yes -	Yes ↑	Yes ↓	Yes ↓	Yes ↓	Yes ↓		
Mhaidat e t al [21]	MSP	6 weeks (8h / day)	Wistar, Male, Adult	Yes -	Yes ↑	Yes ↓		Yes ↓	Yes ↓		Yes - (TBARS)

Alzoubi et al [22]	MSP	8 weeks (8h / day)	Wistar, Male, Adult	Yes -	Yes ↑	Yes ↓	Yes ↓	Yes ↓	Yes -		Yes - (TBARS)
Alzoubi et al [23]	MSP	8 weeks (8h / day)	Wistar, Male, Adult	Yes -	Yes ↑	Yes ↓	Yes ↓	Yes ↓			

SD: sleep deprivation; MSP: multiple small platforms; CP: classical platform/ inverted flowerpot; Srec: sleep recovery; PND: postnatal day; GSH: Reduced Glutathione; GSSG: Oxidized Glutathione; GPx: Glutathione peroxidase; CAT: Catalase; SOD: Superoxide dismutase; NOx: Nitric oxide; MDA: Malonaldehyde; TBARS: Thiobarbituric acid reactive substances; 4- HNE: 4-Hydroxynonenal; *: dentate gyrus, hippocampus; "↑ / ↓ ": significantly increased/ decreased; "-": not significantly increased or decreased; empty cell: determination was not performed

Table S2. Paradoxical sleep deprivation in cortex and other brain areas

**Reference**	**SD protocol**	**SD duration**	**Rat breed, sex, age**	**Anatomical site**	**GSH**	**GSSG**	**GSH/GSSG ratio**	**GPx**	**CAT**	**SOD**	**Nitric oxide**	**Lipid peroxidation**
Mohammed et al [24]	MSP	48 h	Wistar, Male, Adult	Cortex	Yes -						Yes - (NOx)	Yes - (MDA)
Turan et al [5]	MSP	72 h	Wistar, Male, Adult	Prefrontal cortex	Yes -							Yes - (MDA)
Roubalová et al [6]	MSP	72 h	Wistar, Male	Forebrain cortex								Yes ↑ (MDA)
Vosahlikova et al [8]	MSP	72 h	Wistar, Male	Forebrain cortex								Yes ↑ (MDA)
Kwon et al [10]	CP	4 days	Wistar, Male, 5 weeks	Cortex								Yes ↑ (4-HNE)
Suganya et al [25]	MSP	7 days	Wistar, Male, 8 weeks	Cortex	Yes ↓			Yes ↓	Yes ↓	Yes ↓		Yes ↑ (LP)
Turan et al [5]	MSP	72 h	Wistar, Male, Adult	Cerebellum, Brainstem	Yes -							Yes - (MDA)

Zhang et al [26]	MSP	72 h	Sprague-Dawley, Adult	Amygdala						Yes ↓		Yes ↑ (MDA)
Anis Syahirah et al [27]	CP	72 h	Sprague-Dawley, Male, 8-10 weeks	Thalamus	Yes ↓				Yes ↓	Yes ↓		Yes ↑ (MDA)
Ahmad et al [28]	MSP	5 days	Sprague-Dawley, Male and Female, Adult	Whole brain					Yes ↓	Yes ↓		Yes ↑ (MDA)
Jameie et al [29]	CP	6 days	Wistar, Male, Adult	Locus coeruleus	Yes ↓							

SD: sleep deprivation; MSP: multiple small platforms; CP: classical platform/ inverted flowerpot; Srec: sleep recovery; PND: postnatal day; GSH: Reduced Glutathione; GSSG: Oxidized Glutathione; GPx: Glutathione peroxidase; CAT: Catalase; SOD: Superoxide dismutase; NOx: Nitric oxide; MDA: Malonaldehyde; LP: Lipid peroxidation; 4- HNE: 4-Hydroxynonenal; "↑ / ↓ ": significantly increased/ decreased; "-": not significantly increased or decreased; empty cell: determination was not performed

Table S3. Paradoxical sleep deprivation in serum/ plasma

**Reference**	**SD protocol**	**SD duration**	**Rat breed, sex, age**	**GSH**	**GSSG**	**GSH/GSSG ratio**	**GPx**	**CAT**	**SOD**	**Nitric oxide**	**Lipid peroxidation**
Olayaki et al [30]	MSP	20 h	ns								Yes ↑ (MDA)
Solanki et al [31]	MSP	24 h	Long-Evans, Male, Old								Yes ↑ (8-isoprostane)
Hao et al [32]	CP	24, 36, 48 h	Sprague-Dawley, Male, 6 months						Yes ↓		Yes ↑ (MDA)
Zhang et al [33]	CP	48 h	Sprague-Dawley, Male, 6 weeks						Yes ↓		Yes ↑ (MDA)
Hamed et al [34]	MSP	72 h	Wistar, Male, 10 weeks							Yes ↓ (NOx)	

Zhang et al [26]	MSP	72 h	Sprague-Dawley, Adult						Yes ↓		Yes ↑ (MDA)
Rizk et al [35]	MSP	5 days	Wistar, Male								Yes ↑ (MDA)
Olayaki et al [30]	MSP	5 days (20h/day)<br><br>5 days (20h/day) + 5 days Srec	ns								Yes ↑ (MDA)<br><br>Yes - (MDA)
Andrabi et al [13]	CP	6 days (48h SD, 48h Srec, 48h SD, 48h Srec, 48h SD)	Wistar, Male, Adult					Yes -			Yes - (MDA)
Lasisi et al [36]	MSP	7 days (20h/ day) and 7 days continuous	Wistar, Male					Yes – / Yes ↓	Yes –		Yes ↑ (MDA)
Gu et al [37]	MSP	7 days	Wistar, Male				Yes ↓		Yes ↓		
Yildirim et al [38]	MSP	21 days (18h/ day)	Wistar, Male, Adult							Yes - (eNOS)	

SD: sleep deprivation; MSP: multiple small platforms; CP: classical platform/ inverted flowerpot; Srec: sleep recovery; PND: postnatal day; GSH: Reduced Glutathione; GSSG: Oxidized Glutathione; GPx: Glutathione peroxidase; CAT: Catalase; SOD: Superoxide dismutase; NOx: Nitric oxide; eNOS: Endothelial Nitric Oxide Synthase; MDA: Malonaldehyde; ns: not specified; "↑ / ↓ ": significantly increased/ decreased; "-": not significantly increased or decreased; empty cell: determination was not performed

Table S4. Paradoxical sleep deprivation in other non-brain sites

**Reference**	**SD protocol**	**SD duration**	**Rat breed, sex, age**	**Anatomical site**	**GSH**	**GSSG**	**GSH/GSSG ratio**	**GPx**	**CAT**	**SOD**	**Nitric oxide**	**Lipid peroxidation**
Hamed et al [34]	MSP	72 h	Wistar, Male, 10 weeks	Testes, Epididymis	Yes ↓			Yes ↓	Yes ↓	Yes ↓		Yes ↑ (MDA)
Rizk et al [35]	MSP	5 days	Wistar, Male	Testes	Yes ↓			Yes ↓				Yes ↑ (MDA)

Medubi et al [39]	MSP	14 days (20h/ day)	Sprague-Dawley, Male, 12 weeks	Testes	Yes ↓				Yes ↓	Yes ↑		Yes ↑ (MDA)
Siervo et al [40]	MSP	21 days (18h / day)	Wistar, Male, Peripubertal	Testes	Yes -	Yes -	Yes -					Yes ↑ (LP)
Siervo et al [41]	MSP	21 days (18h/ day)	Wistar, Male, Peripubertal	Epididymis caput, cauda	Yes ↑							Yes ↑ (LP)
Hernández Santiago et al [42]	MSP	4 days<br>8 days<br>8 days + 20 days Srec	Wistar, Male, 3 months	Liver, Pancreas					Yes –<br>Yes ↓ / Yes –<br>Yes –	Yes - / Yes ↑<br>Yes ↓ / Yes –<br>Yes ↓ / Yes –		Yes - / Yes ↑ (LP)<br>Yes ↑ / Yes - (LP)<br>Yes ↑ (LP)
			Wistar, Male, 14 months	Liver, Pancreas					Yes ↓ / Yes –<br>Yes ↓, Yes –<br>Yes –	Yes –<br>Yes – / Yes ↑<br>Yes - / Yes ↑		Yes – (LP)<br>Yes – / Yes ↑ (LP)<br>Yes – (LP)
Han et al [43]	MSP	21 days (14h/ day)	Wistar, Male, Adult	Liver						Yes ↓		Yes ↑ (MDA)
Li et al [44]	CP	21 days (18h/ day)<br>21 days (22h/ day)	Wistar, Male, Adult	Liver				Yes –<br>Yes –		Yes –<br>Yes ↓		Yes - (MDA)<br>Yes ↑ (MDA)
Nawi et al [45]	CP	72h<br>72h + 72h Srec	Sprague-Dawley, Male, 8-10 weeks	Aorta						Yes ↓<br>Yes -		Yes ↑ (MDA)<br>Yes – (MDA)
Jiang et al [46]	CP	5 days	Sprague-Dawley, Male, 24 weeks	Aorta							Yes ↓ (NOx)<br>Yes - (eNOS)<br>Yes ↓ (p-eNOS)	
Lasisi et al [36]	MSP	7 days (20h/day) and 7 days continuous	Wistar, Male	Saliva<br>Submandibular					Yes -<br>Yes -	Yes -<br>Yes ↓		Yes - (MDA)<br>Yes - / Yes ↑ (MDA)

Li et al [47]	CP	21 days (18h /day)	Wistar, Male	Thyroid								Yes ↑ (MDA)
Roubalová et al [6]	MSP	72 h	Wistar, Male	Kidney<br>Erythrocytes								Yes - (MDA)<br>Yes ↑ (MDA)
Mônico-Neto et al [48]	MSP	4 days	Wistar, Male, 3 months	Plantar muscle<br>Soleus muscle								Yes - (MDA)<br>Yes ↑ (MDA)

SD: sleep deprivation; MSP: multiple small platforms; CP: classical platform/ inverted flowerpot; Srec: sleep recovery; PND: postnatal day; GSH: Reduced Glutathione; GSSG: Oxidized Glutathione; GPx: Glutathione peroxidase; CAT: Catalase; SOD: Superoxide dismutase; NOx: Nitric oxide; eNOS: Endothelial Nitric Oxide Synthase; p-eNOS: phosphorylated Endothelial Nitric Oxide Synthase; MDA: Malonaldehyde; LP: Lipid peroxidation; "↑ / ↓ ": significantly increased/ decreased; "-": not significantly increased or decreased; empty cell: determination was not performed

Table S5. Total sleep deprivation in brain areas

Reference	SD protocol	SD duration	Rat breed, sex, age	Anatomical site	GSH	GSSG	GSH/GSSG ratio	GPx	CAT	SOD	Nitric oxide	Lipid peroxidation
Forouzanfar et al [49]	GH	6 h	Wistar, Male, 10 weeks	Hippocampus								Yes - (MDA)
Bajaj et al [50]	GH	12 h	Wistar, Female, 13-15 months	Hypothalamus							Yes ↓ (nNOS)	
Tang et al [51]	ASD	14 days	Wistar, Male	Cortex and Hippocampus	Yes ↓				Yes ↓	Yes ↓		Yes ↑ (LP)
Chang et al [52]	DOW	5 days SD + 2 days Srec (3 total cycles) + 3 months Srec	Wistar, Male, Weanling	Hippocampus				Yes ↓	Yes ↓	Yes ↓ / -		

SD: sleep deprivation; Srec: sleep recovery; GH: Gentle handling; ASD: Automated sleep deprivation; DOW: Disk over water; GSH: Reduced Glutathione; GSSG: Oxidized Glutathione; GPx: Glutathione peroxidase; CAT: Catalase; SOD: Superoxide dismutase; nNOS: neuronal Nitric Oxide Synthase; MDA: Malonaldehyde; LP: lipid peroxidation; "↑ / ↓ ": significantly increased/ decreased; "-": not significantly increased or decreased; empty cell: determination was not performed

Table S6. Total sleep deprivation in non-brain areas

Reference	SD protocol	SD duration	Rat breed, sex, age	Anatomical site	GSH	GSSG	GSH/GSSG ratio	GPx	CAT	SOD	Nitric oxide	Lipid peroxidation
Renn et al [53]	DOW	5 days	Wistar, Male	Liver				Yes ↓	Yes ↓	Yes ↓		Yes ↑ (MDA)
Chen et al [54]	DOW	5 days SD + 2 days Srec (3 total cycles)	Wistar, Male, Adult	Liver				Yes ↓	Yes ↓	Yes ↓		Yes ↑ (MDA)
Atrooz et al [55]	ASD	14 days<br>6h/ day - 1st week<br>8h/ day - 2nd week	Sprague-Dawley, Male, PND 19	Plasma								PND 33: Yes ↑ (8-isoprostane)<br><br>PND 90: Yes - (8-isoprostane)

SD: sleep deprivation; Srec: sleep recovery; ASD: Automated sleep deprivation; DOW: Disk over water; PND: Postnatal day; GSH: Reduced Glutathione; GSSG: Oxidized Glutathione; GPx: Glutathione peroxidase; CAT: Catalase; SOD: Superoxide dismutase; MDA: Malonaldehyde; "↑ / ↓ ": significantly increased/ decreased; "-": not significantly increased or decreased; empty cell: determination was not performed

Table S7. Gene expression in paradoxical and total sleep deprivation

Reference	SD protocol	SD duration	Rat breed, sex, age	Anatomical site	GSH	GSSG	GSH/GSSG ratio	GPx	CAT	SOD	Nitric oxide	Lipid peroxidation
Alvarenga et al [56]	PSD - MSP	96h<br><br>21 days (18h / day)	Wistar-Hannover, Male, Adult	Testes							Yes ↑ (iNOS)<br>Yes ↓ (eNOS)<br><br>Yes ↑ (iNOS)<br>Yes - (eNOS)	
Atrooz et al [55]	TSD - ASD	14 days<br>6h/ day - 1st week<br>8h/ day - 2nd week	Sprague-Dawley, Male, PND 19	Prefrontal cortex				PND 33: Yes ↑<br>PND 90: Yes -	PND 33: Yes -<br>PND 90: Yes -	PND 33: Yes ↑<br>PND 90: Yes -		

SD: sleep deprivation; PSD: paradoxical sleep deprivation; TSD: total sleep deprivation; MSP: multiple small platforms; ASD: automated sleep deprivation; PND: postnatal day; GSH: Reduced Glutathione; GSSG: Oxidized Glutathione; GPx: Glutathione peroxidase; CAT: Catalase; SOD: Superoxide dismutase; eNOS: Endothelial Nitric Oxide Synthase; iNOS: Inducible nitric oxide synthase; "↑ / ↓ ": significantly increased/ decreased; "-": not significantly increased or decreased; empty cell: determination was not performed

References

1. Tricco, A.C.; Lillie, E.; Zarin, W.; O'Brien, K.K.; Colquhoun, H.; Levac, D.; Moher, D.; Peters, M.D.J.; Horsley, T.; Weeks, L.; et al. PRISMA Extension for Scoping Reviews (PRISMA-ScR): Checklist and Explanation. *Ann. Intern. Med.* **2018**, *169*, 467–473.
2. Villafuerte, G.; Miguel-Puga, A.; Rodríguez, E.M.; Machado, S.; Manjarrez, E.; Arias-Carrión, O. Sleep Deprivation and Oxidative Stress in Animal Models: A Systematic Review. *Oxid. Med. Cell. Longev.* **2015**, *2015*, 234952.
3. Sahin, L.; Cevik, O.S.; Cevik, K.; Guven, C.; Taskin, E.; Kocahan, S. Mild Regular Treadmill Exercise Ameliorated the Detrimental Effects of Acute Sleep Deprivation on Spatial Memory. *Brain Res.* **2021**, *1759*, 147367.
4. Aboul Ezz, H.S.; Noor, A.E.; Mourad, I.M.; Fahmy, H.; Khadrawy, Y.A. Neurochemical Effects of Sleep Deprivation in the Hippocampus of the Pilocarpine-Induced Rat Model of Epilepsy. *Iran. J. Basic Med. Sci.* **2021**, *24*, 85–91.
5. Turan, I.; Sayan Ozacmak, H.; Ozacmak, V.H.; Ergenc, M.; Bayraktaroğlu, T. The Effects of Glucagon-like Peptide 1 Receptor Agonist (Exenatide) on Memory Impairment, and Anxiety- and Depression-like Behavior Induced by REM Sleep Deprivation. *Brain Res. Bull.* **2021**, *174*, 194–202.
6. Roubalová, L.; Vošahlíková, M.; Slaninová, J.; Kaufman, J.; Alda, M.; Svoboda, P. Tissue-Specific Protective Properties of Lithium: Comparison of Rat Kidney, Erythrocytes and Brain. *Naunyn. Schmiedebergs. Arch. Pharmacol.* **2021**, *394*, 955–965.
7. Zuo, J.-X.; Li, M.; Jiang, L.; Lan, F.; Tang, Y.-Y.; Kang, X.; Zou, W.; Wang, C.-Y.; Zhang, P.; Tang, X.-Q. Hydrogen Sulfide Prevents Sleep Deprivation-Induced Hippocampal Damage by Upregulation of Sirt1 in the Hippocampus. *Front. Neurosci.* **2020**, *14*, 169.
8. Vosahlikova, M.; Roubalova, L.; Cechova, K.; Kaufman, J.; Musil, S.; Miksik, I.; Alda, M.; Svoboda, P. Na+/K+-ATPase and Lipid Peroxidation in Forebrain Cortex and Hippocampus of Sleep-Deprived Rats Treated with Therapeutic Lithium Concentration for Different Periods of Time. *Prog. Neuropsychopharmacol. Biol. Psychiatry* **2020**, *102*, 109953.
9. Nabaee, E.; Kesmati, M.; Shahriari, A.; Khajehpour, L.; Torabi, M. Cognitive and Hippocampus Biochemical Changes Following Sleep Deprivation in the Adult Male Rat. *Biomed. Pharmacother.* **2018**, *104*, 69–76.
10. Kwon, K.J.; Lee, E.J.; Kim, M.K.; Jeon, S.J.; Choi, Y.Y.; Shin, C.Y.; Han, S.-H. The Potential Role of Melatonin on Sleep Deprivation-Induced Cognitive Impairments: Implication of FMRP on Cognitive Function. *Neuroscience* **2015**, *301*, 403–414.
11. Zhang, Q.; Su, G.; Zhao, T.; Wang, S.; Sun, B.; Zheng, L.; Zhao, M. The Memory Improving Effects of Round Scad (Decapterus Maruadsi) Hydrolysates on Sleep Deprivation-Induced Memory Deficits in Rats via Antioxidant and Neurotrophic Pathways. *Food Funct.* **2019**, *10*, 7733–7744.
12. Gou, X.-J.; Cen, F.; Fan, Z.-Q.; Xu, Y.; Shen, H.-Y.; Zhou, M.-M. Serum and Brain Metabolomic Variations Reveal Perturbation of Sleep Deprivation on Rats and Ameliorate Effect of Total Ginsenoside Treatment. *Int. J. Genomics Proteomics* **2017**, *2017*, 5179271.
13. Andrabi, M.; Andrabi, M.M.; Kunjunni, R.; Sriwastva, M.K.; Bose, S.; Sagar, R.; Srivastava, A.K.; Mathur, R.; Jain, S.; Subbiah, V. Lithium Acts to Modulate Abnormalities at Behavioral, Cellular, and Molecular Levels in Sleep Deprivation-Induced Mania-like Behavior. *Bipolar Disord.* **2020**, *22*, 266–280.
14. Konakanchi, S.; Raavi, V.; Ml, H.K.; Shankar Ms, V. Effect of Chronic Sleep Deprivation and Sleep Recovery on Hippocampal CA3 Neurons, Spatial Memory and Anxiety-like Behavior in Rats. *Neurobiol. Learn. Mem.* **2022**, *187*, 107559.
15. Alzoubi, K.H.; Al-Jamal, F.F.; Mahasneh, A.F. Cerebrolysin Prevents Sleep Deprivation Induced Memory Impairment and Oxidative Stress. *Physiol. Behav.* **2020**, *217*, 112823.

16. Alzoubi, K.H.; Al Mosabih, H.S.; Mahasneh, A.F. The Protective Effect of Edaravone on Memory Impairment Induced by Chronic Sleep Deprivation. *Psychiatry Res.* **2019**, *281*, 112577.
17. Alzoubi, K.H.; Mayyas, F.A.; Khabour, O.F.; Bani Salama, F.M.; Alhashimi, F.H.; Mhaidat, N.M. Chronic Melatonin Treatment Prevents Memory Impairment Induced by Chronic Sleep Deprivation. *Mol. Neurobiol.* **2016**, *53*, 3439–3447.
18. Massadeh, A.M.; Alzoubi, K.H.; Milhem, A.M.; Rababa'h, A.M.; Khabour, O.F. Evaluating the Effect of Selenium on Spatial Memory Impairment Induced by Sleep Deprivation. *Physiol. Behav.* **2022**, *244*, 113669.
19. Alzoubi, K.H.; Rababa'h, A.M.; Owaisi, A.; Khabour, O.F. L-Carnitine Prevents Memory Impairment Induced by Chronic REM-Sleep Deprivation. *Brain Res. Bull.* **2017**, *131*, 176–182.
20. Alzoubi, K.H.; Khabour, O.F.; Albawaana, A.S.; Alhashimi, F.H.; Athamneh, R.Y. Tempol Prevents Chronic Sleep-Deprivation Induced Memory Impairment. *Brain Res. Bull.* **2016**, *120*, 144–150.
21. Mhaidat, N.M.; Alzoubi, K.H.; Khabour, O.F.; Tashtoush, N.H.; Banihani, S.A.; Abdul-razzak, K.K. Exploring the Effect of Vitamin C on Sleep Deprivation Induced Memory Impairment. *Brain Res. Bull.* **2015**, *113*, 41–47.
22. Alzoubi, K.H.; Mayyas, F.; Abu Zamzam, H.I. Omega-3 Fatty Acids Protects against Chronic Sleep-Deprivation Induced Memory Impairment. *Life Sci.* **2019**, *227*, 1–7.
23. Alzoubi, K.H.; Malkawi, B.S.; Khabour, O.F.; El-Elimat, T.; Alali, F.Q. Arbutus Andrachne L. Reverses Sleep Deprivation-Induced Memory Impairments in Rats. *Mol. Neurobiol.* **2018**, *55*, 1150–1156.
24. Mohammed, H.S.; Khadrawy, Y.A. Electrophysiological and Neurochemical Evaluation of the Adverse Effects of REM Sleep Deprivation and Epileptic Seizures on Rat's Brain. *Life Sci.* **2021**, *273*, 119303.
25. Suganya, K.; Kayalvizhi, E.; Yuvaraj, R.; Chandrasekar, M.; Kavitha, U.; Konakanchi Suresh, K. Effect of Withania Somnifera on the Antioxidant and Neurotransmitter Status in Sleep Deprivation Induced Wistar Rats. *Bioinformation* **2020**, *16*, 631–637.
26. Zhang, L.; Guo, H.-L.; Zhang, H.-Q.; Xu, T.-Q.; He, B.; Wang, Z.-H.; Yang, Y.-P.; Tang, X.-D.; Zhang, P.; Liu, F.-E. Melatonin Prevents Sleep Deprivation-Associated Anxiety-like Behavior in Rats: Role of Oxidative Stress and Balance between GABAergic and Glutamatergic Transmission. *Am. J. Transl. Res.* **2017**, *9*, 2231–2242.
27. Anis Syahirah, M.S.; Che Badariah, A.A.; Idris, L.; Rosfaiizah, S.; Liza, N. Impact of Rapid Eye Movement Sleep Deprivation on Pain Behaviour and Oxidative Stress in the Thalamus: Role of Tualang Honey Supplementation. *Malays. J. Med. Sci.* **2022**, *29*, 69–79.
28. Ahmad, L.; Mujahid, M.; Mishra, A.; Rahman, M.A. Protective Role of Hydroalcoholic Extract of Cajanus Cajan Linn Leaves against Memory Impairment in Sleep Deprived Experimental Rats. *J. Ayurveda Integr. Med.* **2020**, *11*, 471–477.
29. Jameie, S.B.; Mesgar, S.; Aliaghaei, A.; Raoofi, A.; Amini, M.; Khodagholi, F.; Danyali, S.; Sorraya, M.; Sadeghi, Y. Neuroprotective Effect of Exogenous Melatonin on the Noradrenergic Neurons of Adult Male Rats' Locus Coeruleus Nucleus Following REM Sleep Deprivation. *J. Chem. Neuroanat.* **2019**, *100*, 101656.
30. Olayaki, L.A.; Sulaiman, S.O.; Anoba, N.B. Vitamin C Prevents Sleep Deprivation-Induced Elevation in Cortisol and Lipid Peroxidation in the Rat Plasma. *Niger. J. Physiol. Sci.* **2015**, *30*, 5–9.
31. Solanki, N.; Atrooz, F.; Asghar, S.; Salim, S. Tempol Protects Sleep-Deprivation Induced Behavioral Deficits in Aggressive Male Long-Evans Rats. *Neurosci. Lett.* **2016**, *612*, 245–250.
32. Hao, L.; Yang, R.; Xu, H. Effects of Exercise of Equal Intensity on Working Memory and BDNF Protein Expression in the Prefrontal Cortex in Rats with Different Degrees of Sleep Deprivation. *Int. J. Clin. Exp. Med.* **2019**, *12*, 11490–11496.
33. Zhang, L.; Tang, M.; Wang, F.; Zhao, Y.; Liu, Z. The Impact of Different Exercise Intensities on Working Memory and BDNF Protein Expression in Prefrontal Cortex of Sleep Deprivation Rat. *Int. J. Clin. Exp. Med.* **2017**, *10*, 9265–9270.

34. Hamed, M.A.; Akhigbe, T.M.; Akhigbe, R.E.; Aremu, A.O.; Oyedokun, P.A.; Gbadamosi, J.A.; Anifowose, P.E.; Adewole, M.A.; Aboyeji, O.O.; Yisau, H.O.; et al. Glutamine Restores Testicular Glutathione-Dependent Antioxidant Defense and Upregulates NO/CGMP Signaling in Sleep Deprivation-Induced Reproductive Dysfunction in Rats. *Biomed. Pharmacother.* **2022**, *148*, 112765.
35. Rizk, N.I.; Rizk, M.S.; Mohamed, A.S.; Naguib, Y.M. Attenuation of Sleep Deprivation Dependent Deterioration in Male Fertility Parameters by Vitamin C. *Reprod. Biol. Endocrinol.* **2020**, *18*, 2.
36. Lasisi, T.J.; Shittu, S.-T.T.; Abeje, J.I.; Ogunremi, K.J.; Shittu, S.A. Paradoxical Sleep Deprivation Induces Oxidative Stress in the Submandibular Glands of Wistar Rats. *J. Basic Clin. Physiol. Pharmacol.* **2021**, *33*, 399–408.
37. Gu, X.; Zhang, S.; Ma, W.; Wang, Q.; Li, Y.; Xia, C.; Xu, Y.; Zhang, T.; Yang, L.; Zhou, M. The Impact of Instant Coffee and Decaffeinated Coffee on the Gut Microbiota and Depression-Like Behaviors of Sleep-Deprived Rats. *Front. Microbiol.* **2022**, *13*, 778512.
38. Yildirim, G.; Ozcan, K.M.; Keskin, O.; Tekeli, F.; Kaymaz, A.A. Effects of Chronic REM Sleep Deprivation on Lipocalin-2, Nitric Oxide Synthase-3, Interleukin-6 and Cardiotrophin-1 Levels: An Experimental Rat Model. *Sleep Biol. Rhythms* **2019**, *17*, 305–310.
39. Medubi, L.J.; Nwosu, N.C.; Medubi, O.O.; Lawal, O.R.; Ama, C.; Kusemiju, T.O.; Osinubi, A.A. Increased de Novo Glutathione Production Enhances Sexual Dysfunctions in Rats Subjected to Paradoxical Sleep Deprivation. *JBRA Assist Reprod* **2021**, *25*, 215–222.
40. Siervo, G.E.M.L.; Ogo, F.M.; Staurengo-Ferrari, L.; Anselmo-Franci, J.A.; Cunha, F.Q.; Cecchini, R.; Guarnier, F.A.; Verri, W.A., Jr; Fernandes, G.S.A. Sleep Restriction during Peripuberty Unbalances Sexual Hormones and Testicular Cytokines in Rats. *Biol. Reprod.* **2019**, *100*, 112–122.
41. Siervo, G.E.M.L.; Ogo, F.M.; Valério, A.D.; Silva, T.N.X.; Staurengo-Ferrari, L.; Alvarenga, T.A.; Cecchini, R.; Verri, W.A.; Guarnier, F.A.; Andersen, M.L.; et al. Sleep Restriction in Wistar Rats Impairs Epididymal Postnatal Development and Sperm Motility in Association with Oxidative Stress. *Reprod. Fertil. Dev.* **2017**, *29*, 1813–1820.
42. Hernández Santiago, K.; López-López, A.L.; Sánchez-Muñoz, F.; Cortés Altamirano, J.L.; Alfaro-Rodríguez, A.; Bonilla-Jaime, H. Sleep Deprivation Induces Oxidative Stress in the Liver and Pancreas in Young and Aging Rats. *Heliyon* **2021**, *7*, e06466.
43. Han, C.; Li, F.; Liu, Y.; Ma, J.; Yu, X.; Wu, X.; Zhang, W.; Li, D.; Chen, D.; Dai, N.; et al. Modified Si-Ni-San Decoction Ameliorates Central Fatigue by Improving Mitochondrial Biogenesis in the Rat Hippocampus. *Evid. Based. Complement. Alternat. Med.* **2018**, *2018*, 9452127.
44. Li, Y.; Zhang, Y.; Ji, G.; Shen, Y.; Zhao, N.; Liang, Y.; Wang, Z.; Liu, M.; Lin, L. Autophagy Triggered by Oxidative Stress Appears to Be Mediated by the AKT/MTOR Signaling Pathway in the Liver of Sleep-Deprived Rats. *Oxid. Med. Cell. Longev.* **2020**, *2020*, 6181630.
45. Nawi, A.; Eu, K.L.; Faris, A.N.A.; Wan Ahmad, W.A.N.; Noordin, L. Lipid Peroxidation in the Descending Thoracic Aorta of Rats Deprived of REM Sleep Using the Inverted Flowerpot Technique. *Exp. Physiol.* **2020**, *105*, 1223–1231.
46. Jiang, J.; Gan, Z.; Li, Y.; Zhao, W.; Li, H.; Zheng, J.-P.; Ke, Y. REM Sleep Deprivation Induces Endothelial Dysfunction and Hypertension in Middle-Aged Rats: Roles of the ENOS/NO/CGMP Pathway and Supplementation with L-Arginine. *PLoS One* **2017**, *12*, e0182746.
47. Li, Y.; Zhang, W.; Liu, M.; Zhang, Q.; Lin, Z.; Jia, M.; Liu, D.; Lin, L. Imbalance of Autophagy and Apoptosis Induced by Oxidative Stress May Be Involved in Thyroid Damage Caused by Sleep Deprivation in Rats. *Oxid. Med. Cell. Longev.* **2021**, *2021*, 5645090.
48. Mônico-Neto, M.; Lee, K.S.; da Luz, M.H.M.; Pino, J.M.V.; Ribeiro, D.A.; Cardoso, C.M.; Sueur-Maluf, L.L.; Tufik, S.; Antunes, H.K.M. Histopathological Changes and Oxidative Damage in Type I and Type II Muscle Fibers in Rats Undergoing Paradoxical Sleep Deprivation. *Cell. Signal.* **2021**, *81*, 109939.

49. Forouzanfar, F.; Gholami, J.; Foroughnia, M.; Payvar, B.; Nemati, S.; Khodadadegan, M.A.; Saheb, M.; Hajali, V. The Beneficial Effects of Green Tea on Sleep Deprivation-Induced Cognitive Deficits in Rats: The Involvement of Hippocampal Antioxidant Defense. *Heliyon* **2021**, *7*, e08336.
50. Bajaj, P.; Kaur, G. Acute Sleep Deprivation-Induced Anxiety and Disruption of Hypothalamic Cell Survival and Plasticity: A Mechanistic Study of Protection by Butanol Extract of Tinospora Cordifolia. *Neurochem. Res.* **2022**, *47*, 1692–1706.
51. Tang, H.; Li, K.; Dou, X.; Zhao, Y.; Huang, C.; Shu, F. The Neuroprotective Effect of Osthole against Chronic Sleep Deprivation (CSD)-Induced Memory Impairment in Rats. *Life Sci.* **2020**, *263*, 118524.
52. Chang, H.-M.; Lin, H.-C.; Cheng, H.-L.; Liao, C.-K.; Tseng, T.-J.; Renn, T.-Y.; Lan, C.-T.; Chen, L.-Y. Melatonin Successfully Rescues the Hippocampal Molecular Machinery and Enhances Anti-Oxidative Activity Following Early-Life Sleep Deprivation Injury. *Antioxidants (Basel)* **2021**, *10*, doi:10.3390/antiox10050774.
53. Renn, T.-Y.; Yang, C.-P.; Wu, U.-I.; Chen, L.-Y.; Mai, F.-D.; Tikhonova, M.A.; Amstislavskaya, T.G.; Liao, W.-C.; Lin, C.-T.; Liu, Y.-C.; et al. Water Composed of Reduced Hydrogen Bonds Activated by Localized Surface Plasmon Resonance Effectively Enhances Anti-Viral and Anti-Oxidative Activities of Melatonin. *Chem. Eng. J.* **2022**, *427*, 131626.
54. Chen, H.-C.; Cheng, C.-Y.; Chen, L.-Y.; Chang, C.-C.; Yang, C.-P.; Mai, F.-D.; Liao, W.-C.; Chang, H.-M.; Liu, Y.-C. Plasmon-Activated Water Effectively Relieves Hepatic Oxidative Damage Resulting from Chronic Sleep Deprivation. *RSC Adv.* **2018**, *8*, 9618–9626.
55. Atrooz, F.; Liu, H.; Kochi, C.; Salim, S. Early Life Sleep Deprivation: Role of Oxido-Inflammatory Processes. *Neuroscience* **2019**, *406*, 22–37.
56. Alvarenga, T.A.; Hirotsu, C.; Mazaro-Costa, R.; Tufik, S.; Andersen, M.L. Impairment of Male Reproductive Function after Sleep Deprivation. *Fertil. Steril.* **2015**, *103*, 1355-62.e1.
